# How does transient signaling input affect the spike timing of postsynaptic neuron near the threshold regime: an analytical study

**DOI:** 10.1007/s10827-017-0664-6

**Published:** 2017-12-01

**Authors:** Safura Rashid Shomali, Majid Nili Ahmadabadi, Hideaki Shimazaki, Seyyed Nader Rasuli

**Affiliations:** 1 School of Cognitive Sciences, Institute for Research in Fundamental Sciences (IPM), P.O. Box 19395-5746 (1954851167), Tehran, Iran; 2 Control and Intelligent Processing Center of Excellence, School of Electrical and Computer Engineering, College of Engineering, University of Tehran, Tehran, 14395-515 Iran; 3 Graduate School of Informatics, Kyoto University, Yoshida-honmachi, Sakyo-ku, Kyoto, 606-8501 Japan; 4 Honda Research Institute Japan, Honcho 8-1, Wako-shi, Saitama 351-0188 Japan; 5 Department of Physics, University of Guilan, Rasht, 41335-1914 Iran; 6 School of Physics, Institute for Research in Fundamental Sciences (IPM), P.O. Box 19395-5531, Tehran, Iran

**Keywords:** First-passage time density, Transient signaling input, Strong synapse, Gaussian noise, Threshold regime, Fisher information

## Abstract

The noisy threshold regime, where even a small set of presynaptic neurons can significantly affect postsynaptic spike-timing, is suggested as a key requisite for computation in neurons with high variability. It also has been proposed that signals under the noisy conditions are successfully transferred by a few strong synapses and/or by an assembly of nearly synchronous synaptic activities. We analytically investigate the impact of a transient signaling input on a leaky integrate-and-fire postsynaptic neuron that receives background noise near the threshold regime. The signaling input models a single strong synapse or a set of synchronous synapses, while the background noise represents a lot of weak synapses. We find an analytic solution that explains how the first-passage time (ISI) density is changed by transient signaling input. The analysis allows us to connect properties of the signaling input like spike timing and amplitude with postsynaptic first-passage time density in a noisy environment. Based on the analytic solution, we calculate the Fisher information with respect to the signaling input’s amplitude. For a wide range of amplitudes, we observe a non-monotonic behavior for the Fisher information as a function of background noise. Moreover, Fisher information non-trivially depends on the signaling input’s amplitude; changing the amplitude, we observe one maximum in the high level of the background noise. The single maximum splits into two maximums in the low noise regime. This finding demonstrates the benefit of the analytic solution in investigating signal transfer by neurons.

## Introduction

High variability in spiking activities of *in vivo* cortical neurons is considered as one of the fundamentals of information processing by networks of neurons (Softky and Koch 1993; Shadlen and Newsome 1998). Since it is difficult to experimentally control mechanisms that underlie the highly variable neuronal activity, theoretical and computational analysis of a stochastically spiking neuron model are invaluable approaches to investigate how information is transferred via the variable spiking activities (Abbott et al. 2012). Statistics of spike timing beyond the spike-rate conveys information in the sensory systems; in particular, neuron’s first-spike time after stimulus onset can encode most of the information in the sensory cortex (Petersen et al. 2001; Panzeri et al. 2001; Van Rullen and Thorpe 2001; Furukawa and Middlebrooks 2002; Johansson and Birznieks 2004; Van Rullen et al. 2005). Hence the spike-timing distribution, if attained at sufficient accuracy, could be a building block in modeling neural computation (Herz et al. 2006); it would explain consequences of activity-dependent plasticity (Babadi and Abbott 2013), information transmission by a population of neurons (Silberberg et al. 2004; De La Rocha et al. 2007; Pitkow and Meister 2012) and even behavior (Pitkow et al. 2015). An analytical solution would serve this purpose; however, the non-linear dynamics of a single neuron has so far prevented obtaining such a solution.

The variability observed in spike-timing is thought to reflect fluctuations of synaptic inputs rather than the intrinsic noise of neurons (Mainen and Sejnowski 1995). A neuron is sensitive to input fluctuations and fires irregularly if inputs from excitatory and inhibitory neurons are balanced at levels near but below the threshold (Shadlen and Newsome 1998). Intracellular recordings from *in vivo* cortical neurons have revealed ubiquity of such balanced inputs from excitatory and inhibitory populations (Wehr and Zador 2003; Okun and Lampl 2008). The balanced inputs are self-organized in sparsely connected networks with relatively strong synaptic connections and result in asynchronous population activities (van Vreeswijk and Sompolinsky 1996; 1998; Kumar et al. 2008; Renart et al. 2010). Encouragingly, a recent experiment (Tan et al. 2014) demonstrated that membrane potential of macaque V1 neurons are dynamically clamped *near the threshold* when a stimulus is presented to the animal. All these evidence place importance on developing an analytic solution to understand neural behavior near the threshold regime.

On the other hand, the distribution of synaptic strength is typically a log-normal distribution, which indicates the presence of a few extremely strong synapses and a majority of weak synapses (Song et al. 2005; Lefort et al. 2009; Ikegaya et al. 2013; Buzsáki and Mizuseki 2014; Cossell et al. 2015). These strong synapses may form signaling inputs (Abbott et al. 2012), with the aid of other weak synapses (Song et al. 2005; Teramae et al. 2012; Ikegaya et al. 2013; Cossell et al. 2015). Moreover, it has been long debated that nearly synchronized inputs, from multiple neurons, act as a strong signal on top of the noisy background input (Stevens and Zador 1998; Diesmann et al. 1999; Salinas and Sejnowski 2001; Takahashi et al. 2012). The strong input, in many cases, is a short lasting signal. For example, the signaling inputs which code stimulus information in early sensory processing areas like in primary visual and auditory cortex, are usually known to be transient (Gollisch and Herz 2005; Geisler et al. 2007). Thus, besides many weak synapses which form a noisy background input, we should also consider strong or temporally coordinated synaptic events which induce *strong transient signaling inputs*.

The leaky integrate-and-fire (LIF) neuron model is the simplest model that captures the important features of cortical neurons (Rauch et al. 2003; La Camera et al. 2004; Jolivet et al. 2008). This simple model is largely used to investigate the input-output relation of postsynaptic neuron (Tuckwell 1988; Brunel and Sergi 1998; Burkitt and Clark 1999; Lindner et al. 2005; Burkitt 2006a, 2006b; Richardson 2007, 2008; Richardson and Swarbrick 2010; Helias et al. 2013; Iolov et al. 2014). There are analytical studies which obtained the linear response of the neuron to oscillatory signaling input (Bulsara et al. 1996; Brunel and Hakim 1999; Brunel et al. 2001; Lindner and Schimansky-Geier 2001), excitatory and inhibitory synaptic jumps (Richardson and Swarbrick 2010; Helias et al. 2010) or transient input (Herrmann and Gerstner 2001; Helias et al. 2010, 2011). However, a closed-form analytical solution for the impact of *strong transient signaling input* on a LIF neuron model subject to Gaussian noise is not achieved yet.

Here we analytically derive the first-passage time density of a LIF neuron receiving transient signaling input with arbitrary amplitude; the background input is noisy but balanced at the threshold regime. We extend our solution for the arbitrary shape of transient signaling input. As an application of this solution, we calculate the Fisher information with respect to input’s amplitude; the maximum of Fisher information provides the minimum error to estimate the signaling input’s amplitude from spiking activity. We quantify the noise level and signal’s amplitude which yield the best possible discrimination.

## Results

### Impact of a transient signaling input on postsynaptic spiking

**Fig. 1:**
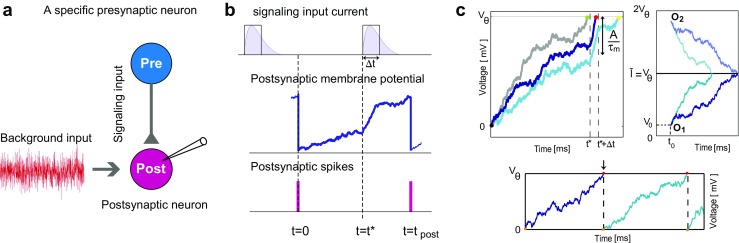
The framework to study the impact of a specified presynaptic neuron on postsynaptic spiking activity. **a** The effect of specified presynaptic neuron is separated from the noisy background input, which is produced by the rest of presynaptic neurons. **b** A schematic view of the signaling input activity and its effect on the membrane potential and spiking of the postsynaptic neuron. **c** Postsynaptic membrane potential versus time, Top-left: There can be different trajectories, due to the noise. Any trajectory, if not reached the threshold before signaling input arrival at *t*^∗^, shows a sharp increase during *t*^∗^to *t*^∗^ + Δ*t* time window. Top-right: For the particular case of $\bar {I}=V_{\theta }$, we can use the image method. The membrane potential trajectory and its mirror image coincide at $V=\bar {I}$ line. This can help to fulfill the thresholding criteria if it is the same line of *V* = *V*_*𝜃*_. Bottom: It shows the membrane potential trajectory of the postsynaptic neuron. When the voltage reaches to *V*_*𝜃*_, the neuron fires and the voltage resets to 0. Here we particularly consider the first spike after *t* = 0

We consider a specified presynaptic neuron that provides the transient signaling input, while the rest of presynaptic neurons produce noisy background inputs to the postsynaptic neuron (Fig. 1a). The question is how a single spike of the signaling input affects the spike timing of the postsynaptic neuron (Fig. 1b). We use the LIF neuron model for postsynaptic neuron; the membrane potential, *V*, evolves with:

1 $$\begin{array}{@{}rcl@{}} \tau_{\mathrm{m}} \frac{d V}{dt} = - (V - V_{r}) + I(t), \end{array} $$

where *τ*_m_ is the membrane time constant, and *I*(*t*)is the total input current. The neuron produces a spike when its voltage reaches the threshold, *V*_*𝜃*_. The membrane voltage then resets to its resting value, *V*_*r*_, which we assume to be zero without loss of generality.

The effect of specified signaling input, and the rest of excitatory/inhibitory presynaptic neurons come through the input current *I*(*t*):

2 $$\begin{array}{@{}rcl@{}} I(t) = I_{0}(t) + {\Delta} I(t, t^{*}), \end{array} $$

where *I*_0_(*t*) is the background input induced by presynaptic neurons, and Δ*I*(*t*,*t*^∗^) is the signaling input from the specified neuron; here *t*^∗^represents the time that the signaling input arrives. For large number of presynaptic neurons, the uncorrelated background input is approximated as: $I_{0}(t) = \bar {I} + \xi (t)$, where $\bar {I}$ is the mean input strength and *ξ*(*t*) is a *zero mean* Gaussian noise (i.e. < *ξ*(*t*) >= 0and < *ξ*(*t*)*ξ*(*t*^′^) >= 2*Dδ*(*t* − *t*^′^), where *δ*(*t*) is the Dirac delta function).

When a spike from presynaptic neuron arrives at synaptic terminal, the postsynaptic current instantaneously increases according to the strength of the synapse, and decays with a time constant of *τ*_*s*_. For *τ*_*s*_ ≪ *τ*_m_ which is measured for fast currents generated by *AMPA* and *GABA*_*A*_receptors (Destexhe et al. 1998), we can model the input current as (Stern et al. 1992):

3 $$\begin{array}{@{}rcl@{}} {\Delta} I(t, t^{*})=A \times \left\{\begin{array}{ll} 0 &{\qquad\qquad} t < t^{*},\\ 1/{\Delta} t & {\qquad\qquad}t^{*} \leq t \leq t^{*}+{\Delta} t,\\ 0 & {\qquad\qquad} t^{*}+{\Delta} t < t, \end{array}\right. \end{array} $$

where Δ*t* ∼ *τ*_*s*_. This resembles a current which begins at *t* = *t*^∗^, remains constant for a short time window, *t* ∈ [*t*^∗^,*t*^∗^ + Δ*t*], and vanishes after that. It transmits a net charge of *A* regardless of Δ*t*; it also converges to the Dirac delta function in the limit of Δ*t* → 0(i.e. Δ*I*(*t*,*t*^∗^) → *A* *δ*(*t* − *t*^∗^)). In this limit, the process (see Eq. (1)) converges to jump-diffusion process (Kou and Wang 2003).

As mentioned, a key element of this article is to predict when the postsynaptic neuron spikes if the signaling input arrives at *t*^∗^. We consider the last spike of the postsynaptic neuron as the origin of time, *t* = 0, and *analytically* predict when the *first postsynaptic spike* will happen (Fig. 1b). However, because of the stochastic term in the background input, *ξ*(*t*), we cannot predict the exact time of the next spike, but can describe its probability density, *J*(*V*_*𝜃*_,*t*).

We consider an ensemble of many postsynaptic neurons (or many repetitions of the same experiment with a single postsynaptic neuron); the life of each neuron in this ensemble is described by a trajectory of *V* (*t*), which is governed by the LIF equation (see Fig. 1c, top-left). All trajectories begin from the same point (*V* = 0 at *t* = 0), but do not follow the same path; because of different values realized for the stochastic background noise, *ξ*(*t*). The neuron initiates a spike if a trajectory passes the threshold voltage, *V*_*𝜃*_. Then *J*(*V*_*𝜃*_,*t*), which we shortly call *first-passage time density*, is the probability density function that a trajectory passes *V*_*𝜃*_ at time *t*. However, to obtain *J*(*V*_*𝜃*_,*t*), we have to know *P*(*V*,*t*), the probability density that a trajectory has the potential *V* at time *t*. This *membrane potential probability density* satisfies the Fokker-Planck (FP) equation (Risken 1984; Kardar 2007):

4 $$\begin{array}{@{}rcl@{}} \frac{\partial}{\partial t}P(V,t)&=&\frac{D}{{\tau_{\mathrm{m}}}^{2}}\frac{\partial^ 2}{\partial V^{2}}P(V,t)\\ &&+\frac{1}{\tau_{\mathrm{m}}}\frac{\partial}{\partial V}[(V-\bar{I}-{\Delta} I(t, t^{*}))\,P(V,t)]. \end{array} $$

Here, the temporal evolution of *P*(*V*,*t*)is governed by (i) a diffusion term which is a signature of the stochastic input (i.e. *ξ*(*t*)), and (ii) a drift term which represents both the leak and the non-stochastic currents (i.e. $\bar {I}$ and Δ*I*(*t*,*t*^∗^)).

Threshold nonlinearity of neuronal spike generation is dictated as a boundary condition in Eq. (4). The LIF neuron *spikes* if it passes the threshold voltage. Since each membrane trajectory ends at the threshold, there is no neuron with *V* > *V*_*𝜃*_. In the continuum limit, this results in the *absorbing boundary condition* of *P*(*V* ≥ *V*_*𝜃*_,*t*) = 0 (Gerstner et al. 2014). Here, we do not consider the reappearance of the trajectory from the resting potential after each spike occurs; because we are interested in neuron’s first spike only (Fig. 1c, down). This results in the absorbing boundary condition instead of the widely used periodic boundary condition to derive firing rate (Brunel and Hakim 1999; Brunel et al. 2001; Lindner and Schimansky-Geier 2001; Richardson and Swarbrick 2010).

Finally, we will obtain *P*(*V*,*t*)for *t* > *t*_0_ by solving Eq. (4) under this boundary condition once we specify an initial distribution of the membrane potential at time *t* = 0. Here we use *P*(*V*,0) = *δ*(*V* ) as we assumed that all membrane trajectories started from *V* = 0.

Unfortunately, the analytical solution of *P*(*V*,*t*)and consequently *J*(*V*_*𝜃*_,*t*)is not attainable in general even if we discard the signaling input from the equation. However, we may obtain the analytical solution at a particular regime known as the *threshold regime*, which will be described in detail as follows. For a fixed noise strength (i.e., *D*), a simple ratio $\bar {I}/V_{\theta }$ determines how *P*(*V*,*t*) and the corresponding first-passage time density of *J*(*V*_*𝜃*_,*t*) behave. The neuron regularly spikes if $\bar {I}$ significantly exceeds *V*_*𝜃*_; because the high value of mean input robustly drives neuron to its threshold. If, on the other hand, $\bar {I} \ll V_{\theta }$, there would be occasional spikes whenever the noise or the signaling input happens to be strong enough to prevail the gap between $\bar {I}$ and *V*_*𝜃*_. An interesting regime exists somewhere in-between; for $\bar {I} \simeq V_{\theta }$, a modest signaling input or some conventional noise can induce spike of the postsynaptic neuron. This is the near *threshold regime*. It was empirically observed (Shadlen and Newsome 1998; Tan et al. 2014) and suggested as a basis of high variability in neural networks (van Vreeswijk and Sompolinsky 1996, 1998). The quest for a *closed-form analytical solution* for Eq. (4) also leads us to the very same regime. There exists a closed-form solution for *P*(*V*,*t*), and consequently for *J*(*V*_*𝜃*_,*t*), if (i) $\bar {I}=V_{\theta }$ and (ii) no signaling input is applied: Δ*I*(*t*,*t*^∗^) = 0(Wang and Uhlenbeck 1945; Sugiyama et al. 1970; Bulsara et al. 1996). Below, we describe the reason behind this peculiarity of the threshold regime and revisit the closed-form solution without the signaling input. We then extend this analytical solution to include effect of the signaling input.

### The first-passage time density in the absence of signaling input

We begin with the simpler condition in which the signaling input is turned off (Δ*I*(*t*,*t*^∗^) = 0). Even in such a case, solutions for *P*(*V*,*t*)are, in general, available only in a non-closed form such as inverse Laplace transforms (Siegert 1951; Ricciardi and Sato 1988; Ostojic 2011). However, there exists a closed-form analytical solution for the particular case of the threshold regime, $\bar {I}=V_{\theta }$ (Wang and Uhlenbeck 1945; Sugiyama et al. 1970; Tuckwell 1988).

A closed-form solution for probability density would exist for arbitrary $\bar {I}$ if we could neglect the absorbing boundary condition. Assume that we have freed ourselves from the absorbing boundary condition, and that the membrane potential has a definite value of *V* = *V*_0_ at time *t* = *t*_0_, there exists a closed-form analytical solution for Eq. (4), for *t* > *t*_0_. It would be the *free Green’s function*, and it reads (Uhlenbeck and Ornstein 1930):

5 $$\begin{array}{@{}rcl@{}} G_{f}{\kern-.5pt}({\kern-.7pt}V\!,{\kern-.8pt}t{\kern-.5pt};\! V_{0}{\kern-.5pt},{\kern-.8pt}t_{0}{\kern-.5pt})\! &=&\! \sqrt{\frac{\tau_{\mathrm{m}}}{2 \pi D} \frac{1}{1-r^{2}(t-t_{0})}}\\ &&\times {\kern-.8pt} \exp\!\left[\! -\frac{\tau_{\mathrm{m}}}{2D} \frac{({\kern-.5pt}V\! -\!\bar{I}\! -\! ({\kern-.5pt}V_{0}\! -\! \bar{I}) r({\kern-.9pt}t\! -\! t_{0}){\kern-.5pt})^{2}}{1-r^{2}(t-t_{0})}{\kern-.5pt}\right].\\ \end{array} $$

Here *V*_0_ and *t*_0_ quote the *initial condition*, and *r*(*t*) = exp[−*t*/*τ*_m_]. The free Green’s function describes a probability density of the membrane trajectories which all begin from the point **O**_1_ = (*t*_0_,*V*_0_), but follow different paths due to the noise (see Fig. 1c, Right). Since we have neglected the threshold for the moment, the trajectories do not end as they pass the threshold line, *V* = *V*_*𝜃*_. Thus we can freely consider any initiating point, even above the threshold line, for the trajectories. It would be $\textbf {O}_{2}=(t_{0},2\bar {I}-V_{0})$, the mirror-image point of **O**_1_, with respect to the $V=\bar {I}$ line (Fig. 1c, Right). The probability density for this initiating point is $G_{f}(V,t;\,2\bar {I}-V_{0},t_{0})$. The encouraging fact is that the two Green’s functions yield equal values on the mirror-line: $G_{f}(V=\bar {I},t;\,V_{0},t_{0})=G_{f}(V=\bar {I},t;\,2\bar {I}-V_{0},t_{0})$. Conclusively, we define the main Green’s function as:

6 $$\begin{array}{@{}rcl@{}} G{\kern-.3pt}({\kern-.3pt}V{\kern-.3pt},{\kern-.3pt}t{\kern-.3pt};{\kern-.3pt}\,V_{0}{\kern-.3pt},{\kern-.3pt}t_{0})\! =\! G_{f}({\kern-.3pt}V{\kern-.3pt},{\kern-.3pt}t{\kern-.3pt};{\kern-.3pt}\,V_{0}{\kern-.3pt},{\kern-.3pt}t_{0}){\kern-.3pt}-{\kern-.3pt}G_{f}(V,t;\,2\bar{I}-V_{0},t_{0}).\!\!\!\!\!\!\\ \end{array} $$

This linear combination of the free Green’s functions, satisfies the linear Fokker-Planck equation (i.e., Eq. (4)). It also approaches zero on the $V=\bar {I}$ line. Now, if we choose $\bar {I}$ equal to *V*_*𝜃*_, this means that *G*(*V*,*t*; *V*_0_,*t*_0_)also satisfies the *absorbing boundary condition*. Thus we can utilize the analytical free Green’s functions to obtain an analytical solution under the absorbing boundary condition only at the threshold regime.

For our main problem, in which the postsynaptic neuron has the certain voltage of *V* = 0 at *t* = 0, the probability density of membrane potential is simply:

7 $$\begin{array}{@{}rcl@{}} P_{0}(V,t)=G(V,t;\,0,0). \end{array} $$

The probability of spiking between *t* and *t* + *dt*, is proportional to the number of voltage trajectories which pass the threshold in [*t*,*t* + *dt*] (see Fig. 1c, left). This equals $J_{0}(V,t)|_{V=V_{\theta }}dt$ where *J*_0_(*V*,*t*) is the current density:

8 $$\begin{array}{@{}rcl@{}} J_{0}(V,t)=-\frac{D}{{\tau_{\mathrm{m}}}^{2}} \frac{\partial}{\partial V} P_{0}(V,t)-\frac{V-\bar{I}}{\tau_{\mathrm{m}}}P_{0}(V,t). \end{array} $$

For *V* = *V*_*𝜃*_, Eq. (8) yields the *first-passage time density*; it simplifies to (Wang and Uhlenbeck 1945; Sugiyama et al. 1970; Tuckwell 1988; Bulsara et al. 1996):

9 $$\begin{array}{@{}rcl@{}} J_{0}(V_{\theta},t)&=&\frac{1}{\tau_{\mathrm{m}}}\sqrt{\frac{2}{\pi}\frac{\tau_{\mathrm{m}} V_{\theta}^{2}}{ D }\frac{r^{2}(t)}{ (1-r^{2}(t))^{3}}} \\ &&\times \exp{\left[-\frac{{\tau_{\mathrm{m}} V_{\theta}}^{2}}{2 D}\frac{ r^{2}(t)}{1-r^{2}(t)}\right]}, \end{array} $$

where *r*(*t*) = exp[−*t*/*τ*_m_]. Apart from a 1/*τ*_m_ pre-factor in Eq. (9), which stands for its time inverse dimensionality, the overall shape of function is characterized by a dimensionless ratio of *D*/(*τ*_m_*V𝜃*2). The ratio quantifies the strength of input background noise relative to the other competing factors. Other relevant quantities are also characterized by this ratio. For example, the maximum value of first-passage time density, *J*_0_(*V*_*𝜃*_,*t*), occurs at $t_{\max }=\tau _{\mathrm {m}}\,{\textbf {h}}(\,D/(\tau _{\mathrm {m}} V_{\theta }^{2})\,)$ where:

10 $$\begin{array}{@{}rcl@{}} {\textbf{h}} (x)=\frac{1}{2}\ln\left( \frac{1}{2x}\left( 1-x+\sqrt{9x^{2}-2x + 1}\right)\right). \end{array} $$

For weak enough noise (i.e. $x=D/(\tau _{\mathrm {m}} V_{\theta }^{2}) \ll 1$), this function simplifies to $0.5\times \ln (\tau _{\mathrm {m}} V_{\theta }^{2}/D)$. Finally, it is important to extend this formalism to more plausible sub/supra-threshold cases. In Appendix I, we show how a scaling approach does help us to do so.

### The transient signaling input modifies the probability density and first-passage time density

In this subsection, we extend the above analysis to the case in which signaling input is additionally applied to the neuron. The presence of the signaling input modifies *P*(*V*,*t*) and consequently *J*(*V*_*𝜃*_,*t*). To obtain a clear causal picture, we rewrite Eq. (4) as:

11 $$\begin{array}{@{}rcl@{}} \left( \frac{\partial}{\partial t}-\frac{D}{{\tau_{\mathrm{m}}}^{2}}\frac{\partial^ 2}{\partial V^{2}} -\frac{1}{\tau_{\mathrm{m}}} -\frac{V-\bar{I}}{\tau_{\mathrm{m}}}\frac{\partial}{\partial V} \right) P(V,t)\\=-\frac{\Delta I(t, t^{*})}{\tau_{\mathrm{m}}}\frac{\partial}{\partial V}P(V,t). \end{array} $$

We assume that Δ*I*(*t*,*t*^∗^) corrects the initial *threshold regime* answer of *P*_0_(*V*,*t*)to *P*(*V*,*t*) = *P*_0_(*V*,*t*) + Δ*P*(*V*,*t*); *P*_0_(*V*,*t*)is the analytical solution of membrane potential density in the absence of the signaling input, and Δ*P*(*V*,*t*) is the correction, due to the signaling input. Δ*P*(*V*,*t*) would be zero if the signaling input did not exist, *A* = 0 (see Eq. (3)). For arbitrary signaling input, Δ*P*(*V*,*t*)would be a function of *A*, the signaling input’s strength. This lets us write a Taylor series for Δ*P*(*V*,*t*)as:

12 $$\begin{array}{@{}rcl@{}} {\Delta} P(V,t)={\Sigma}_{n = 1}^{n=\infty} \delta P_{n}(V,t), \end{array} $$

where *δP*_*n*_(*V*,*t*) ∝ *A*^*n*^. Since Δ*P*(*V*,*t*) vanishes for *A* = 0, the constant term, *δP*_*n*= 0_(*V*,*t*) ∝ *A*^0^, is not included in the series. We plug $P(V,t)=P_{0}(V,t)+ {\Sigma }_{n = 1}^{n=\infty } \delta P_{n}(V,t)$ into Eq. (11); for each *n*, we consider terms proportional to *A*^*n*^, on both sides of equality, as a separate equation. For *n* = 1the equation reads:

13 $$\begin{array}{@{}rcl@{}} \left( \frac{\partial}{\partial t}-\frac{D}{{\tau_{\mathrm{m}}}^{2}}\frac{\partial^ 2}{\partial V^{2}} -\frac{1}{\tau_{\mathrm{m}}} -\frac{V-\bar{I}}{\tau_{\mathrm{m}}}\frac{\partial}{\partial V} \right)\delta P_{1}(V,t)\\ =-\frac{\Delta I(t, t^{*})}{\tau_{\mathrm{m}}}\frac{\partial}{\partial V}P_{0}(V,t), \end{array} $$

This is the *first-order perturbation* equation, as both its sides are proportional to *A*^1^. To address its boundary conditions, we note that Δ*P*(*V*,*t*)is zero before the occurrence of the specified signaling input (i.e. *t* < *t*^∗^); consequently we obtain *δP*_1_(*V*,*t* < *t*^∗^) = 0. Moreover, the absorbing boundary condition at *V* = *V*_*𝜃*_ results in Δ*P*(*V*_*𝜃*_,*t*) = 0, from which we conclude that *δP*_1_(*V*_*𝜃*_,*t*) = 0. These let us use the aforementioned Green’s function, and write *δP*_1_(*V*,*t*)as a Green’s integral over the source term, the right side of Eq. (13):

14 $$\begin{array}{@{}rcl@{}} \delta P_{1}(V,t)&=& {\int}_{t_{0}= 0}^{t}dt_{0} {\int}_{V_{0}=-\infty}^{V_{\theta}} d V_{0}\,G(V,t;V_{0},t_{0} )\\ && \times \left[ -\frac{\Delta I(t_{0}, t^{*})}{\tau_{\mathrm{m}}}\frac{\partial}{\partial V_{0}} P_{0} (V_{0} ,t_{0} )\right]. \end{array} $$

Equation (14) could be further simplified, if we note that Δ*I*(*t*_0_,*t*^∗^) is zero for *t*_0_ < *t*^∗^ or *t*_0_ > *t*^∗^ + Δ*t*; thus the *t*_0_ in *G*(*V*,*t*;*V*_0_,*t*_0_) and (*∂*/*∂V* )*P*_0_(*V*_0_,*t*_0_) always belong to [*t*^∗^,*t*^∗^ + Δ*t*], a short time interval. As Δ*t* ≪ *τ*_m_, we conclude that the two functions are *almost constant* during this time interval and approximate them with *G*(*V*,*t*;*V*_0_,*t*^∗^) and (*∂*/*∂V* )*P*_0_(*V*_0_,*t*^∗^)respectively. This further simplifies Eq. (14):

15 $$\begin{array}{@{}rcl@{}} \delta P_{1}{\kern-.5pt}(V,t)& = & -{\int}_{t_{0}= 0}^{t} \frac{\Delta I(t_{0}, t^{*})}{\tau_{\mathrm{m}}} dt_{0} \\ &&\times {\int}_{V_{0}=-\infty}^{V_{\theta}}G{\kern-.5pt}({\kern-.5pt}V{\kern-.5pt},{\kern-.5pt}t;{\kern-.5pt}V_{0},{\kern-.5pt}t^{*}) \,\frac{\partial}{\partial V_{0}}P_{0}({\kern-.5pt}V_{0} ,t^{*})\,d V_{0}.\\ \end{array} $$

Taking the time integral in Eq. (15), we have:

16 $$\begin{array}{@{}rcl@{}} \delta P_{1}{\kern-.5pt}({\kern-.5pt}V{\kern-.5pt},{\kern-.5pt}t{\kern-.5pt})\! & = &\! f_{1}(t,t^{*}) \left( \frac{A}{\tau_{\mathrm{m}}}\right) \\ &&\!\times {\int}_{V_{0}=-\infty}^{V_{\theta}}G(V,t;V_{0},t^{*})\,\frac{\partial}{\partial V_{0}}P_{0}(V_{0} ,t^{*})\,d V_{0},\\ \end{array} $$

where

17 $$\begin{array}{@{}rcl@{}} f_{1}(t,t^{*})=-\left\{\begin{array}{l} 0 {\qquad\qquad\qquad\quad}\,\, t < t^{*},\\ (t-t^{*})/{\Delta} t {\qquad\quad} t^{*} \leq t \leq t^{*}+{\Delta} t,\\ 1 {\qquad\qquad\qquad\quad}\,\, t^{*}+{\Delta} t < t. \end{array}\right. \end{array} $$

For *n* ≥ 2, the *nth order perturbation* equation is the same as Eq. (13) by replacing *δP*_1_(*V*,*t*) and *δP*_0_(*V*,*t*) with *δP*_*n*_(*V*,*t*) and *δP*_*n*− 1_(*V*,*t*). A recursive formalism, then, yields:

18 $$\begin{array}{@{}rcl@{}} \delta{\kern-.3pt} P_{n}{\kern-.3pt}({\kern-.3pt}V,{\kern-.3pt}t{\kern-.3pt})\! & = &\! f_{n}(t,t^{*}) \left( \frac{A}{\tau_{\mathrm{m}}}\right)^{n}\\ &&\!\times\! {\int}_{V_{0}=-\infty}^{V_{\theta}}{\kern-.3pt}G{\kern-.3pt}({\kern-.3pt}V{\kern-.3pt},{\kern-.3pt}t{\kern-.3pt};{\kern-.3pt}V_{0},{\kern-.3pt}t^{*}) \,\frac{\partial^{n}}{\partial {V_{0}^{n}}}P_{0}(V_{0} ,t^{*})\,d V_{0},\\ \end{array} $$

where

19 $$\begin{array}{@{}rcl@{}} f_{n}(t,t^{*})=\frac{(-1)^{n}}{n!}\left\{\begin{array}{l} 0 {\qquad\qquad\qquad\qquad} t < t^{*},\\ {[(t-t^{*})/{\Delta} t]}^{n} {\quad\quad}\,\,\, t^{*} \leq t \leq t^{*}+{\Delta} t,\\ 1 {\qquad\qquad\qquad\qquad} t^{*}+{\Delta} t < t. \end{array}\right. \end{array} $$

This helps us to calculate the series in Eq. (12) and obtain Δ*P*(*V*,*t*). For *t* > *t*^∗^ + Δ*t*, for example, it reads:

20 $$\begin{array}{@{}rcl@{}} {\Delta} P(V,t) &=& {\int}_{V_{0}=-\infty}^{V_{\theta}} G(V,t;V_{0},t^{*})\\ &&\times\left[ {\Sigma}_{n = 1}^{n=\infty} \frac{1}{n!} \left( \frac{-A}{\tau_{\mathrm{m}}}\right)^{n} \frac{\partial^{n}}{\partial {V_{0}^{n}}}P_{0}(V_{0} ,t^{*})\right]\,d V_{0} \end{array} $$

The summation in bracket is the Taylor expansion of *P*_0_(*V*_0_ − *A*/*τ*_m_,*t*^∗^) − *P*_0_(*V*_0_,*t*^∗^); thus for *t* > *t*^∗^ + Δ*t*:

21 $$\begin{array}{@{}rcl@{}} {\Delta} P(V,t) &=& {\int}_{V_{0}=-\infty}^{V_{\theta}} G(V,t;V_{0},t^{*})\\ &&\times \left[ P_{0}\left( V_{0}-\frac{A}{\tau_{\mathrm{m}}}\,, t^{*}\right)-P_{0}(V_{0},t^{*})\right ]\,d V_{0}. \end{array} $$

For *t*^∗^≤ *t* ≤ *t*^∗^ + Δ*t*, a similar reasoning yields:

22 $$\begin{array}{@{}rcl@{}} {\Delta}{\kern-.5pt} P{\kern-.5pt}({\kern-.5pt}V{\kern-.5pt},{\kern-.5pt}t{\kern-.5pt})\! & = &\! {\int}_{V_{0}=-\infty}^{V_{\theta}} G(V,t;V_{0},t^{*})\\ &&\times\! \left[\! P_{0}\!\left( \! V_{0}\! -\! \frac{A}{\tau_{\mathrm{m}}}\! \times\! \frac{t\! -\! t^{*}}{\Delta t}\,, t^{*}\!\right)\! -\! P_{0}{\kern-.5pt}\left( {\kern-.5pt}V_{0},t^{*}\right)\!\right]d V_{0}. \end{array} $$

And evidently, for *t* < *t*^∗^, *δP*_*n*_(*V*,*t*) = 0. We use the combination rule, for propagators:

23 $$\begin{array}{@{}rcl@{}} P_{0}(V,t) = {\int}_{V_{0}=-\infty}^{V_{\theta}} G(V,t;V_{0},t_{0}) P_{0}(V_{0}, t_{0}) d V_{0}. \end{array} $$

The final result, *P*(*V*,*t*) = *P*_0_(*V*,*t*) + Δ*P*(*V*,*t*), simplifies as:

24 $$\begin{array}{@{}rcl@{}} P(V, t)=\left\{\begin{array}{l} P_{0}(V, t) {\qquad\qquad\qquad\qquad\qquad\qquad\qquad\qquad\qquad\qquad\quad}\,\,\,\,\, t < t^{*},\\ \\ {\int}_{V_{0}=-\infty}^{V_{\theta}} G(V,t;V_{0},t^{*}) P_{0}\left( V_{0}- {\frac{A}{\tau_{\mathrm{m}}}\times\frac{t-t^{*}}{\Delta t}}\,, t^{*}\right) d V_{0}. {\quad}{\quad}{\quad}\,\,\, t^{*} \leq t \leq t^{*}+{\Delta} t,\\ \\ {\int}_{V_{0}=-\infty}^{V_{\theta}} G(V,t;V_{0},t^{*}) P_{0}\left( V_{0}- {\frac{A}{\tau_{\mathrm{m}}}}\,, t^{*}\right) d V_{0}. {\qquad\qquad\qquad}\,\,\, t^{*}+{\Delta} t < t. \end{array}\right. \end{array} $$

Finally, we use *P*(*V*,*t*)to obtain the corrected first-passage time density in the presence of the signaling input:

25 $$\begin{array}{@{}rcl@{}} J(V_{\theta},t)=-\frac{D}{{\tau_{\mathrm{m}}}^{2}} \frac{\partial}{\partial V} P(V,t)|_{V=V_{\theta}}. \end{array} $$

**Fig. 2:**
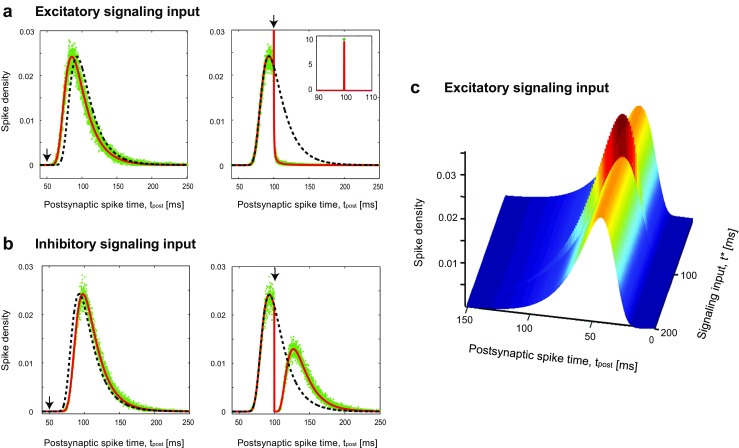
First-passage time density when the neuron receives signaling input and background noise at the threshold regime. The black vertical arrows show when the signaling input arrives; it changes the initial ISI distribution, shown in dashed black curve, to the modified distribution, thick red curve. Even when the signaling input is remarkably strong, the *analytic* modified distribution well matches the simulation results, which come as empty green circles. **a** Left: An early excitatory signaling input at *t*^∗^ = 50 mscauses a leftward temporal shift. Right: A drastic change occurs, If the signaling input arrives at *t*^∗^ = 100 ms, close enough to the peak of initial spiking density. **b** Left: An inhibitory signaling input imposes a mere temporal shift, a delay, if it occurs too early, *t*^∗^ = 50 ms. Right: If the inhibitory signaling input occurs close enough to the peak, $t=t_{\max }$, the spiking density almost divides into two distinct regions. **c** The first-passage time density as a function of the presynaptic spike timing, *t*^∗^, for small excitatory signaling inputs, *A* = 0.2 mV ×ms. The values of the parameters are chosen using the physiologically plausible range (McCormick et al. 1985). Here, we use membrane time constant *τ*_m_ = 20 ms, *A* = 10 mV ms, threshold voltage of *V*_*𝜃*_ = 20 ms, diffusion coefficient of *D* = 0.74 mV^2^msand the simulation time step is Δ*t* = 0.05 ms

While our formalism is applicable to *arbitrary values* of transient signaling input, here we focus on the effect of strong excitatory/inhibitory signaling input on postsynaptic neuron’s response. Figure 1c (top-left) shows how voltage trajectories almost uniformly increase during arrival of excitatory signaling input (i.e. *t* ∈ [*t*^∗^,*t*^∗^ + Δ*t*]). The short period of signal arrival (i.e.Δ*t* ≪ *τ*_m_) guarantees this uniform increase and results in an overall rise of *A*/*τ*_m_. Consequently, if the value of membrane potential of a particular trajectory is larger than *V*_*𝜃*_ − *A*/*τ*_m_ upon signal arrival, it passes the threshold during signal arrival: the neuron fires. If, on the other hand, it is smaller than *V*_*𝜃*_ − *A*/*τ*_m_, the membrane potential does not cross the threshold by the additional signaling input: the neuron does not fire. This simple picture helps us to understand results shown in Fig. 2.

Figure 2a and b show that excitatory and inhibitory signaling inputs can result in quite different spiking behaviors, depending on their arrival time. The dashed-black curve, in both panels of Fig. 2a, b, right and left, shows the first-passage time density in the absence of the signaling input, *J*_0_(*V*_*𝜃*_,*t*). In the left panels, the signaling input arrives at *t*^∗^ = 50 ms; it has *A* = 10 mV × ms which increases or decreases the membrane potential by *A*/*τ*_m_ = 0.5 mV. In this case, the excitatory signaling input shifts the original density leftward for excitation or rightward for inhibition (thick-red-curves). In contrast, the right panels show the spiking density when the signaling input occurs at *t*^∗^ = 100 ms. This is close to $t_{\max }= 0.5\times \tau _{\mathrm {m}}\,\ln (\,\tau _{\mathrm {m}} V_{\theta }^{2}/D\,)\simeq 93~\text {ms}$, at which the *J*_0_(*V*_*𝜃*_,*t*)is maximized. Therefore, when the signal arrives, many trajectories are already close to the threshold potential (i.e., larger than *V*_*𝜃*_ − *A*/*τ*_m_). All such trajectories will spike, due to the aforementioned rise of excitatory *A*/*τ*_m_; this results in a sudden sharp increase of the first-passage time density at *t* = *t*^∗^ (see Fig. 2a, right, and its inset). In contrast, the inhibitory input prevents all trajectories to reach the threshold, which makes a sharp depletion in the first-passage time density (Fig. 2b, right). The effect of inhibitory input fades away after a while and again trajectories approach the threshold; this leads to the second rise of the first-passage time density (Fig. 2b, right). These theoretical predictions were confirmed by numerical simulation of the LIF model (green dots). It is also important to see what will happen if the signaling input comes much later than *t*_max_. This means that most of the trajectories have already reached the threshold voltage, and only a tiny portion of them remains. Consequently, the signaling input does not induce much change in *J*(*V*_*𝜃*_,*t*). In other words, if the signaling input comes too late, the postsynaptic neuron has already fired, and the signal cannot change its first spike-time any more (Fig. 2c). This figure also demonstrates that the first-passage time density undergoes the maximum change when the excitatory input arrives around *t*_max_, the peak time of no-signaling input case.

It is also important to note that there is a critical value for excitatory signal strength for which postsynaptic neuron fires, apart from the signal arrival time. For a signal with *A* ≥ *τ*_m_*V*_*𝜃*_, the aforementioned rise would be *V*_*𝜃*_which results in spiking of almost all trajectories, irrespective of signal arrival time. This introduces another dimensionless ratio of *A*/(*τ*_m_*V*_*𝜃*_), which quantifies the strength of the signaling input.

**Fig. 3:**
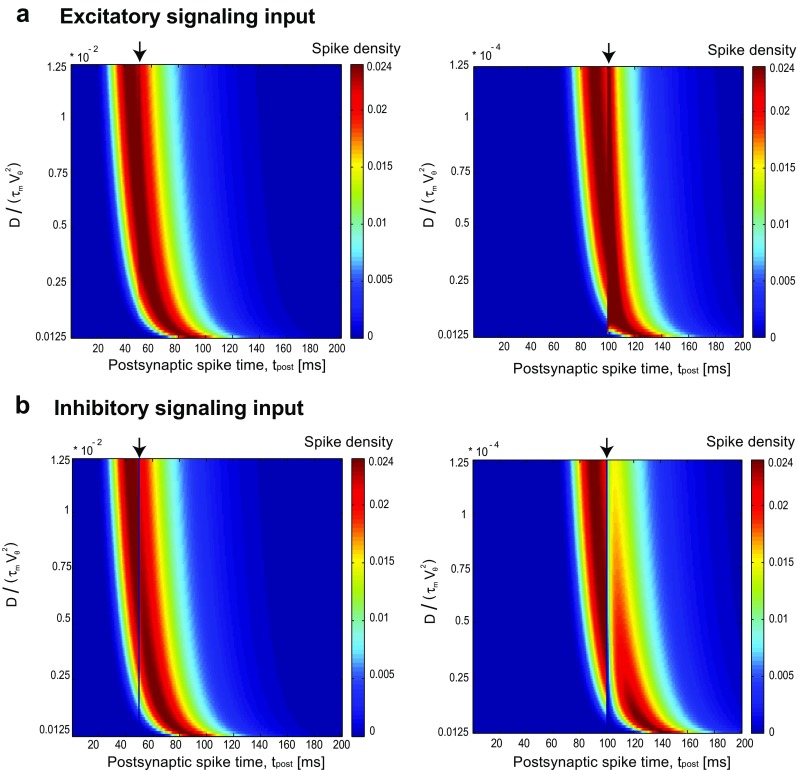
First-passage time density as a function of the scaled diffusion coefficient, *D*/(*τ*_m_*V𝜃*2). **a** Excitatory signaling input: (Left) The signaling input occurs at *t* = 50ms, black arrow. Variation of the scaled diffusion modifies the overall picture of ISI distribution. There is an apparent jump in the spiking density when *D*/(*τ*_m_*V𝜃*2) ≃ 0.25 × 10^− 2^. It is because *D*/(*τ*_m_*V𝜃*2)controls $t_{\max }$, the maximum of the ISI distribution in the absence of the signaling input; and $t_{\max } \simeq 50$ms for *D*/(*τ*_m_*V𝜃*2) ≃ 0.25 × 10^− 2^. For higher/lower values of the scaled diffusion coefficient, however, we see minor modification as $t_{\max }$ would be larger/smaller enough compared to the signaling input arrival time. (Right) The signaling input occurs at *t* = 100 ms; we chose much weaker diffusion coefficient, so that $t_{\max }$ would be comparable to this signaling input arrival time. However, the modification is drastically larger, compared to the left panel. This shows that the influence of the signaling input amplifies, for weaker diffusion/noise strength. **b** The same as in (**a**) but with inhibitory signaling input. Reducing the scaled diffusion coefficient, from left to right, drastically amplifies the modification imposed by the signaling input. The values of the intrinsic parameters are chosen using the physiologically plausible range, *V*_*𝜃*_ = 20 mVand *τ*_m_ = 20 ms(McCormick et al. 1985). The amplitude of signaling input is fixed at *A* = 1 mV ms

Figure 3 shows how the first-passage time density changes with the diffusion coefficient. When the scaled diffusion coefficient increases, the first-passage time density and *t*_max_ shift to the left. So signaling inputs that arrive earlier than *t*_max_at 50 mscan modulate the density (Fig. 3a, left). When the signaling input arrives at time *t*^∗^ = 100 ms which is close to *t*_max_, the first-passage time density is modulated in low diffusion regimes (Fig. 3a, Right). In the case of inhibitory presynaptic spike (Fig. 3b), for high scaled diffusion coefficient, the first-passage time density decreases at the time of presynaptic spike but because of high diffusion, it goes up quickly. As the scaled diffusion decreases, the recovery from inhibition takes time which makes distance between two separated distributions.

**Fig. 4:**
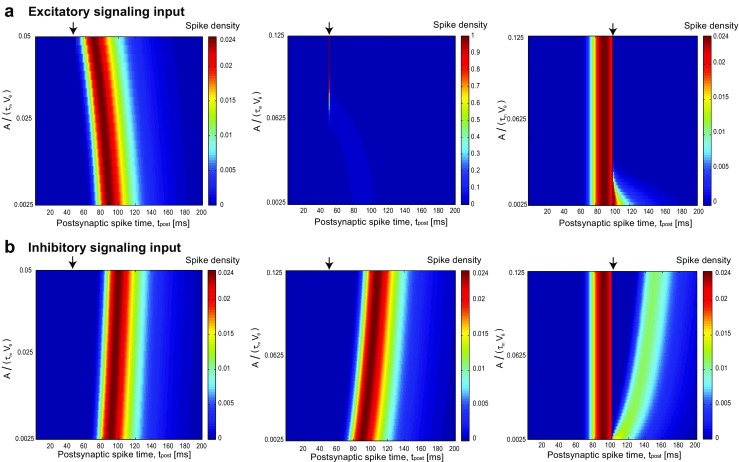
First-passage time density as a function of scaled signaling input amplitude *A*/(*τ*_m_*V*_*𝜃*_). **a** Excitatory signaling input: When the amplitude of signaling input increases, the first-passage time density gradually shifts to the left (**a**, left) and in specific strong amplitude, the density changes to a peak because the signaling input is so strong that makes all the trajectories to fire at the time of signal arrival (**a**, middle). The signaling input arrives at *t*^∗^ = 50 ms(left and middle) and at *t*^∗^ = 100 ms(Right). **b** Inhibitory signaling input: Inhibitory signal arrives at *t*^∗^ = 50 ms(left and middle) and *t*^∗^ = 100 ms(Right). When the input arrival time is much less than $t_{\max }= 90~\text {ms}$, the effect of increasing the amplitude of inhibitory input is just to push the density to the right (**b**, Left and middle). For cases that *t*^∗^is comparable to $t_{\max }$, the first-passage time density breaks at the time of input arrival (**b**, right). As the amplitude increases, the recovery of distribution from that break takes more time and the distance between separated distribution increases nonlinearly. The signaling input arrival is shown by black arrows and the diffusion coefficient is fixed at *D* = 1 mV^2^ms. The values of the intrinsic parameters (*V*_*𝜃*_ = 20 mVand *τ*_m_ = 20 ms) are chosen using the physiologically plausible range (McCormick et al. 1985)

The first-passage time density also depends on the scaled amplitude of the signaling input (Fig. 4). For an excitatory input which fires at time (*t*^∗^) much earlier than *t*_max_, as the amplitude increases, the density moves to the left (Fig. 4a, left) until the amplitude is large enough to make them spike at the same time (Fig. 4a, middle). When inhibitory signaling input arrives near *t*_max_, the density breaks in two parts (Fig. 4b, right). As the amplitude increases, the spiking density is zero not only at the time of presynaptic spiking but also for a duration after that. This duration nonlinearly depends on the strength of signaling input (Fig. 4b, right).

### Arbitrary shapes of the transient signaling input

**Fig. 5:**
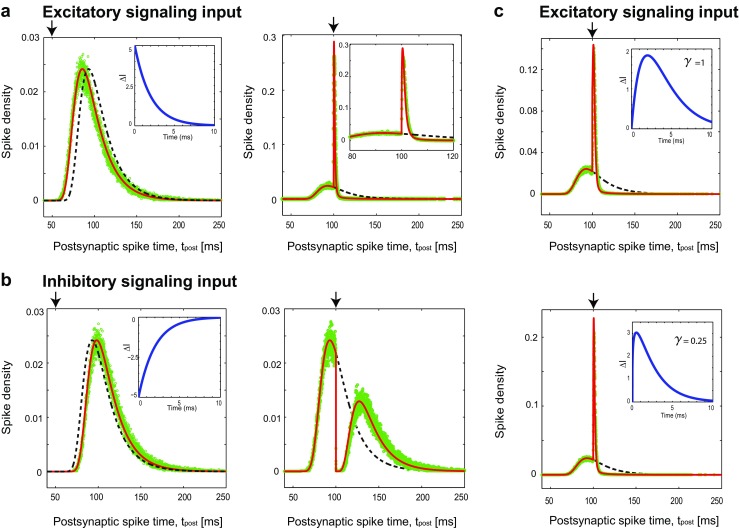
First-passage time density when the neuron receives exponential decaying signaling input and background noise at the threshold regime. The black vertical arrows show when the signaling input arrives; it changes the initial ISI distribution, shown in dashed black curve, to the modified distribution, thick red curve. Even when the signaling input is remarkably strong, the *analytic* modified distribution well matches the simulation results, which come as empty green circles. **a** Left: An early excitatory signaling input at *t*^∗^ = 50 mscauses a leftward temporal shift. The result is very similar to the case of square input (Fig. 2a, left). Right: A drastic change occurs, if the signaling input arrives at *t*^∗^ = 100 ms, close enough to the peak of initial spiking density. The change in density is smoother comparing with Fig. 2a, right. The inset shows the shape of the signaling input. **b** Left: An inhibitory signaling input imposes a mere temporal shift, a delay, if it occurs too early, *t*^∗^ = 50 ms. Right: If the inhibitory signaling input occurs close enough to the peak, $t=t_{\max }$, the spiking density almost divides into two distinct regions. The inset shows the shape of the signaling input. **c** The first-passage time density for the cases that signaling input is gamma function with parameter *γ* = 1(Top) and *γ* = 0.25(Bottom). The values of the parameters are chosen using the physiologically plausible range (McCormick et al. 1985). Here, we use membrane time constant *τ*_m_ = 20 ms, *τ*_*s*_ = 2 ms, *A* = 10 mV ms, threshold voltage of *V*_*𝜃*_ = 20 mV, diffusion coefficient of *D* = 0.74 mV^2^msand the simulation time step is Δ*t* = 0.05 ms

One merit of this formalism is its flexibility to address other shapes of the transient signaling input. We solve the first-passage time problem for the exponentially decaying input, which is more physiologically plausible (i.e. Δ*I*(*t*,*t*^∗^) ∝ exp(−(*t* − *t*^∗^)/*τ*_s_), see Appendix II for the derivation and results). Figure 5a and b show how an exponential transient signaling input modifies the first-passage time density.

For early signaling arrival, the modification due to excitatory (inhibitory) input is a leftward (rightward) shift, very similar to the changes we had for square input (Fig. 2a, b). For an excitatory input at *t*^∗^ = 100 ms (Fig. 5a right) there is a jump upon signal arrival; comparing to the square excitatory input (Fig. 2a right) the jump is less sharp but more wide. Similarly, the inhibitory exponential input at *t*^∗^ = 100 ms induces a fall (Fig. 5b right), similar but less steep than the fall observed for the square inhibitory input (Fig. 2b right). The numerical simulations well verify these analytic results (green circles in Fig. 5a, b).

The analytical solutions for square and exponential input suggest a *general formula* for probability density in the presence of an *arbitrary input current*, Δ*I*(*t*,*t*^∗^), which arrives at *t*^∗^. If the duration of signal arrival is shorter enough than the membrane time constant (i.e. *τ*_s_ ≪ *τ*_m_) we suggest the probability density as:

26 $$\begin{array}{@{}rcl@{}} P(V,t)\,=\,{\int}_{V_{0}=-\infty}^{V_{\theta}} \!G(V,t,V_{0},t^{*})\,P_{0}\left( V_{0}\,-\,\!{{\int}_{0}^{t}}\frac{\Delta I(s,t^{*})}{\tau_{\mathrm{m}}} ds ,\, t^{*}\right)\, \!d V_{0}.\\ \end{array} $$

This conjecture covers our results for both the square input (i.e. Eq. (24)) and the exponential decay (i.e. Eq. (54) in Appendix II). We also test it by comparing the analytical result of first-passage time (obtained using Eq. (26)) with simulation results (Fig. 5c) using the *Gamma function* input current:

27 $$\begin{array}{@{}rcl@{}} {\Delta} I(t,t^{*})\,=\,A\!\times\!\left\{\begin{array}{l} 0 {\qquad\qquad\qquad\qquad\qquad\quad\quad\quad\quad\quad } t < t^{*},\\ {\frac{1}{\Gamma(1+\gamma)}}\left( {\frac{t-t^{*}}{\tau_{\mathrm{s}}}}\right)^{{\gamma}} \!\times\!{\frac{1}{\tau_{\mathrm{s}}}}\,{\exp\left( -\frac{t-t^{*}}{\tau_{\mathrm{s}}}\right)} {\quad}\,\,\,\,\, t^{*} \leq t; \end{array}\right.\\ \end{array} $$

the Γ(*n*) is the Euler’s Gamma function (Davis 1959). The signaling inputs are shown in the insets of Fig. 5c, top (bottom) for *γ* = 1(*γ* = 0.25). The inputs arrive at *t*^∗^ = 100 ms, and have a short duration of *τ*_s_ = 2 ms ≪ *τ*_m_ = 20 ms; we recognize a good agreement between simulation and analytical results. The method also can be extendable to the case that more than one presynaptic spike arrives. The derivation for more spikes of presynaptic neuron comes in Appendix III.

It is worthy to use Eq. (26) and obtain a general solution for the first-passage time density, due to transient signaling input with arbitrary shape. Using Eq. (8), the first-passage time density reads:

28 $$\begin{array}{@{}rcl@{}} J(V_{\theta},t)& = & -\frac{D}{\tau_{\mathrm{m}}^{2}}{\int}_{V_{0}=-\infty}^{V_{\theta}} \tilde{G}(V_{\theta},t,V_0,t^*)\\ &&\times P_{0}\left( V_{0}- {{\int}_{0}^{t}}\frac{\Delta I(s,t^{*})}{\tau_{\mathrm{m}}} ds , t^{*}\right)\,d V_{0}, \end{array} $$

where $\tilde {G}(V_{\theta },t,V_{0},t^{*})=\partial G(V,t,V_{0},t^{*})/\partial V|_{V=V_{\theta }}$; it reads:

29 $$\begin{array}{@{}rcl@{}} \tilde{G}{\kern-.5pt}({\kern-.5pt}V_{\theta},{\kern-.5pt}t,{\kern-.5pt}V_{0},t^{*})&\,=\,&\sqrt{\frac{2 \tau_{\mathrm{m}}^{3}}{\pi D^{3}}} \frac{r(t-t^{*}) \times (V_{0}-V_{\theta})}{(1-r^{2}(t-t^{*}))^{3/2}}\\ &&\times{\kern-.5pt} \exp{\kern-.5pt}{\left[\! -\frac{\tau_{\mathrm{m}}}{2D}\frac{r^{2}(t\,-\,t^{*})\,(V_{0}\,-\,V_{\theta})^{2}}{1-r^{2}(t-t^{*})}\right]}. \end{array} $$

We use Eq. (7) for probability density, *P*_0_(*V* ), and try to calculate the integral in Eq. (28) (see Appendix IV). An expression is derived, for the first-passage time density in the presence of arbitrary signaling input (*τ*_*s*_ ≪ *τ*_m_):

30 $$\begin{array}{@{}rcl@{}} J(V_{\theta},t)&=&\frac{\sqrt{\kappa \, \omega}}{\pi \tau_{\mathrm{m}}} \left\{ \exp\left( -\frac{\varphi_{+}^{2}}{2}\right)\!\left[1+\sqrt{\frac{\pi}{2 \kappa}}\,\varphi_{+}\exp\left( \frac{\varphi_{+}^{2}}{2 \kappa}\right)\right.\times\left( 1+\text{erf}\left( \frac{\varphi_{+}}{\sqrt{2 \kappa}}\right)\right)\right]\\ &&\qquad\quad-\exp\left( -\frac{\varphi_{-}^{2}}{2}\right)\!\left[1\,+\,\sqrt{\frac{\pi}{2 \kappa}}\,\varphi_{-}\exp\!\left( \frac{\varphi_{-}^{2}}{2 \kappa}\right)\left.\times\left( 1+\text{erf}\left( \frac{\varphi_{-}}{\sqrt{2 \kappa}}\right)\right)\right] \right\}, \end{array} $$

where $\text {erf}(x)=(2/\sqrt {\pi }){{\int }_{0}^{x}} \exp (-t^{2})dt$ and *κ*(*t*,*t*^∗^), *ω*(*t*,*t*^∗^) and *φ*_±_(*t*,*t*^∗^)are:

31 $$\begin{array}{@{}rcl@{}} \kappa(t,t^{*}) & \,=\, & (1-r(t)^{2})/(1\,-\,r(t-t^{*})^{2}),\\ \omega(t,t^{*}) & \,=\, & \frac{r(t-t^{*})^{2}(1-r(t^{*})^{2})}{(1-r(t)^{2})^{3}},\\ \varphi_{\pm}(t,t^{*}) & \,=\, & {\sqrt{\frac{\tau_{\mathrm{m}}V_{\mathrm \theta}^{2}}{D(1\,-\,r(t^{*})^{2})}}} \left\{\pm r(t^{*})\,-\,{{\int}_{0}^{t}} \frac{ds}{\tau_{\mathrm{m}}}\frac{\Delta I(s, t^{*})}{V_{\theta}}\right\}. \end{array} $$

The first-passage time density, Eq. (30), is the probability density of the first spike of the postsynaptic neuron when the signaling input arrives at *t*^∗^; the *t*^∗^measures the time elapsed since the *last spike of postsynaptic neuron* up to the *signal arrival*.

There are experimental studies in which the postsynaptic neuron’s spike times both before and after signal arrival are recorded (Blot et al. 2016). In these researches, the *t*^∗^ is observed/assumed. For example studies regarding *timing dependent plasticity*, assume the knowledge of the last postsynaptic spike (Froemke and Dan 2002; Wang et al. 2005). The timing of the last spike may also be learned by intrinsic mechanisms (Johansson et al. 2016; Jirenhed et al. 2017). In such cases, it would be possible to directly apply our formalism to the analysis. However, there are experimental studies which do not take into account such knowledge of the last postsynaptic spike timing (Panzeri et al. 2014); a downstream neuron may have no access to the information of the neuron’s former spikes. Therefore, we should consider all possible values for *t*^∗^, a statistical procedure known as marginalization.

### First spike-timing density after signaling input’s arrival

We observed that timing of the signaling input, relative to the last postsynaptic spike, significantly affects the first-spiking density of the postsynaptic neuron. However, the postsynaptic neuron (as well as downstream neurons) may have no access to this elapsed time. Assume that we monitor the arrival of a signaling input; it would be more convenient to consider arrival time as the time origin. Therefore, we reset the time origin accordingly and ask what is the probability density *f*(*τ*) that postsynaptic neuron fires at *τ* after signaling input arrival.

As a building block to address this question, we need to know the probability of the last postsynaptic spike occurring in a time window between *t*^∗^and *t*^∗^ + *dt*^∗^ before the signal arrival, *P*_back_(*t*^∗^) *dt*^∗^. Following the language of voltage trajectories, this probability can be computed by considering that (A) a trajectory has begun from *V*_r_ = 0in the mentioned time window, but (B) it has not yet reached the threshold at *V* = *V*_*𝜃*_. The answer comes as multiplication of probabilities associated with the two conditions (A) and (B):

32 $$\begin{array}{@{}rcl@{}} P_{\text{back}}(t^{*})\,dt^{*}= \lambda\,dt^{*} \times (1 - {\int}_{0}^{t^{*}} J_{0}(V_{\theta},s) \, ds ). \end{array} $$

where $\lambda =({\int }_{0}^{\infty } s J_{0}(V_{\theta },s) \, ds)^{-1}$ is the spike rate of the postsynaptic neuron in the absence of any signaling input and *J*_0_(*V*_*𝜃*_,*s*) is given by Eq. (9). *P*_back_(*t*^∗^)is known as the density function of *backward recurrence* time in the point process theory (Cox 1962).

The next question is which *portion* of the trajectories, addressed above, will reach the threshold at *τ* to *τ* + *dτ*, after signal arrival. This sets a temporal distance of *t*^∗^ + *τ* between the beginning point of the trajectories with *V* = *V*_r_ = 0to their spiking point at *V* = *V*_*𝜃*_. The answer is a conditional probability which reads:

33 $$\begin{array}{@{}rcl@{}} f (\tau | t^{*} ) d\tau = \frac{ J(V_{\theta},\tau + t^{*}) d\tau}{ 1 - {\int}_{0}^{t^{*}} J_{0}(V_{\theta},s) ds }. \end{array} $$

Note that *J*(*V*_*𝜃*_,*τ* + *t*^∗^) is given by Eq. (30), where the signaling input arrives at *t*^∗^, after the last postsynaptic spike. The denominator is a normalization term to achieve ${\int }_{0}^{\infty } f (\tau | t^{*} )d\tau = 1$.

Having *f*(*τ*|*t*^∗^)and *P*_back_(*t*^∗^), we should integrate over all possible values of backward recurrence time, *t*^∗^, to obtain *f*(*τ*):

34 $$\begin{array}{@{}rcl@{}} f(\tau) &=& {\int}_{0}^{\infty} f(\tau|t^{*}) \times P_{\text{back}}(t^{*}) \, dt^{*} \\ &=& \lambda {\int}_{0}^{\infty} J(V_{\theta},\tau + t^{*}) \,dt^{*} . \end{array} $$

The result in Eq. (34) presents the probability density of first-spike timing after input arrival; the time of input arrival (stimulus onset) should be known by some mechanisms in the cortex (Van Rullen et al. 2005; Panzeri et al. 2014).

It is worth to see how *P*_back_(*t*^∗^)successfully connects us to the existing stationary solution for the membrane potential (Brunel and Hakim 1999; Brunel 2000). We should address the probability of finding the membrane potential between *V*_0_ and *V*_0_ + *dV*_0_ at an arbitrary observation time. We split the task into two questions: First, what is the probability that the last postsynaptic spike has happened in a time window of *t*^∗^to *t*^∗^ + *dt*^∗^, before observation time?; Second, what is the conditional probability that voltage trajectories, which initiated from *t*^∗^ before observation time, have a potential *V* ∈ [*V*_0_,*V*_0_ + *dV*_0_]at the time of observation. The answer for the first question is simply given by the probability density of the backward recurrence time. The answer for the second question is a conditional probability:

35 $$\begin{array}{@{}rcl@{}} \tilde{p}(V_{0} | t^{*}) dV_{0}=\frac{P_{0}(V_{0}, t^{*}) dV_{0}}{{\int}_{-\infty}^{V_{\theta}} P_{0}(V, t^{*}) dV}. \end{array} $$

*P*_0_(*V*,*t*^∗^)is given by Eq. (7). The denominator is a normalizing factor to ensure: ${\int }_{-\infty }^{V_{\theta }} \tilde {p}(V_{0} | t^{*}) dV_{0}= 1$. It has the very same origin we mentioned for the denominator in Eq. (33); in fact, it is easy to verify that the two denominators are equal, due to the conservation of probability: ${\int }_{-\infty }^{V_{\theta }} P_{0}(V, t^{*}) dV = 1 - {\int }_{0}^{t^{*}} J_{0}(V_{\theta },s) ds$. We combine the answers of two questions, and obtain the *stationary probability density* as:

36 $$\begin{array}{@{}rcl@{}} P_{\mathrm{s}}(V_{0}) &=& {\int}_{0}^{\infty} \tilde{p}(V_{0} | t^{*}) \times P_{\text{back}}(t^{*}) \, dt^{*} \\ &=& \lambda {\int}_{0}^{\infty} P_{0}(V_{0}, t^{*}) \, dt^{*}. \end{array} $$

**Fig. 6:**
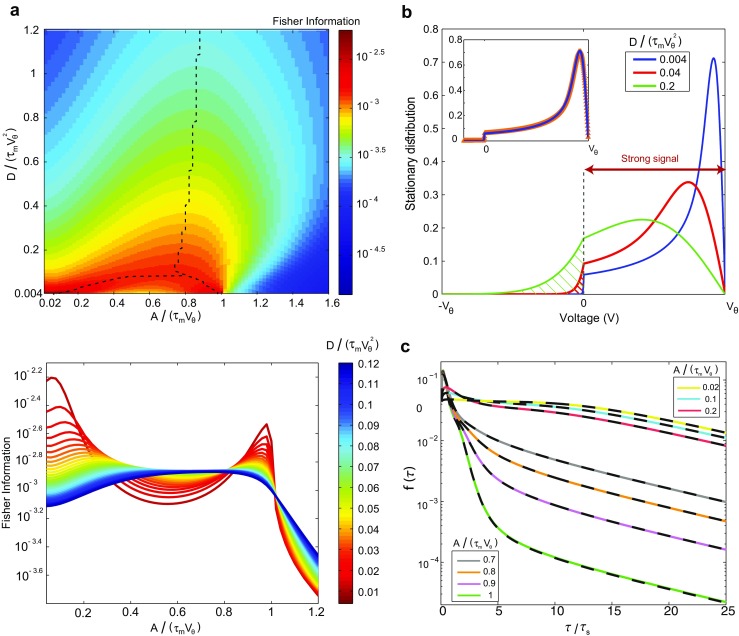
**a**, Top: Fisher information with respect to the amplitude of signaling input in logarithmic scale as a function of the scaled amplitude, *A*/(*τ*_m_*V*_*𝜃*_), and the scaled diffusion coefficient, *D*/(*τ*_m_*V𝜃*2). We use exponential decaying signaling input with *τ*_*s*_ = 1 ms (see Eq. (46)). For high level of the noise, Fisher information has one maximum in strong amplitude, but for a specific level of the noise, it splits into two maximums which occur in strong and weak amplitudes. The dashed black line shows the maximums of Fisher information. It is notable that Fisher information does not monotonically decrease with the noise level; except for *A*/(*τ*_m_*V*_*𝜃*_) ≃ 1and *A*/(*τ*_m_*V*_*𝜃*_) < 0.1, for the rest of scaled amplitudes, the Fisher information is maximized for a certain level of the noise (stochastic resonance). Bottom: in the low noise regime, *D*/(*τ*_m_*V𝜃*2) ≤ 0.088, the Fisher information is maximized either by small signaling input’s amplitudes, *A*/(*τ*_m_*V*_*𝜃*_) < 0.3or by its strong amplitudes, *A*/(*τ*_m_*V*_*𝜃*_) ≃ 1. The two maximums approach each other, as the diffusion coefficient increases, and reach for *D*/(*τ*_m_*V𝜃*2) > 0.088. This one maximum would be robust to the change of the amplitude in a wide range. **b** stationary distribution of the membrane potential for different scaled diffusion coefficient. When strong amplitudes in low diffusion arrive, nearly all trajectories reach the threshold and only small portion of them remains (hatched red area). Inset shows the comparison between stationary distribution from Eqs. (36) and (77) (Brunel and Hakim 1999; Brunel 2000). **c***f*(*τ*)in logarithmic scale for weak and strong signaling inputs: The solid colored lines are obtained using Eq. (34) while the black dashed lines are the results using Eq. (37). The coincidence shows that two equations produce the same result, for the same signaling input’s amplitude. The scaled noise level is *D*/(*τ*_m_*V𝜃*2) = 0.04

Figure 6b-inset shows that *P*_s_(*V*_0_)nicely coincides with the existing stationary solution found by Brunel and Hakim (Brunel and Hakim 1999; Brunel 2000). To use their solution, we have simply put the mean input current equal to the threshold potential, $\bar {I}=V_{\theta }$.

*P*_s_(*V*_0_) provides an alternative approach to find *f*(*τ*). It determines the probability density that the postsynaptic neuron has a membrane potential of *V* = *V*_0_ upon signal arrival, (i.e. *t* = *t*^∗^). We have also obtained how the probability density evolves after signal arrival (i.e. *t* > *t*^∗^), for a square (see Eq. (24)) or exponential (see Eq. (55)) signaling input. We note that the framework of solutions which results in Eq. (24) or Eq. (55) does not depend on the initial choice of *P*_0_(*V*_0_,*t*) or *P*_s_(*V*_0_). Conclusively, if we want to determine first-spiking density, with no previous knowledge about the last postsynaptic spike, we should simply replace *P*_0_(*V*_0_,*t*^∗^) with *P*_s_(*V*_0_) (see Appendix V). This lets us follow our suggestion for *J*(*V*_*𝜃*_,*t*)in the presence of an arbitrary transient input, Eq. (30), and obtain *f*(*τ*)accordingly:

37 $$\begin{array}{@{}rcl@{}} f(\tau)&=&-\frac{D}{\tau_{\mathrm{m}}^{2}}{\int}_{V_{0}=-\infty}^{V_{\theta}} \tilde{G}(V_{\theta},\tau,V_{0},0)\\ &&\times P_{\mathrm{s}}\left( V_{0}- {\int}_{0}^{\tau}\frac{\Delta I(s,0)}{\tau_{\mathrm{m}}} ds\right)\,d V_{0}, \end{array} $$

where $\tilde {G}(V_{\theta },\tau ,V_{0},0)$ is given by Eq. (29). In Fig. 6c, we depict *f*(*τ*)using both Eq. (37), dashed lines, and Eq. (34), full lines. There is a nice coincidence between two sets of curves which shows the consistency of the result from two approaches mentioned here. The result arises from each one of two approaches, has its own advantage. Since Eq. (34) has just one temporal integral, and *J*(*V*_*𝜃*_,*τ*)is already well simplified in Eq. (30), it is computationally easier and faster to work with. At first glance, Eq. (37) also has one temporal integral, however, there is another integral in *P*_s_(*V* ) to reach stationary solution (see Eq. (36)). Consequently, it would be computationally faster to use Eq. (34) but Eq. (37) provides more intuition about how *f*(*τ*)behaves.

### Fisher information

The analytical first-spiking density after input arrival, Eq. (34), allows us to quantify the *minimum error* for any unbiased estimator to decode signaling input’s properties such as its amplitude (input’s strength). Based on Cramer-Rao’s inequality (Rao 1973), the Fisher information provides the lower bound of the estimator’s variance ($\sigma ^{2}_{\text {est}}\geq 1/\mathcal {I}_{FI} $). Applied to spike timing density, maximizing the Fisher information gives us the minimum error to decode an input parameter (e.g. signal’s amplitude) using the spiking activity. Spike timing of postsynaptic neuron contains information that spike count does not carry (Rieke et al. 1999; van Vreeswijk 2001; Toyoizumi et al. 2006). Indeed, discarding spike timing information (specifically first-spike timing) leads to loss of information (Panzeri et al. 2001). Hence here, we investigate the Fisher information based on the spike timing with respect to the strength of signaling input. In this scenario, the decoder must know the input arrival time and the first spike time after that. It was discussed that this knowledge about the input arrival time as a time reference may be known by, for example, network oscillations or other mechanisms in cortical/sensory systems (Van Rullen et al. 2005; Panzeri et al. 2014). Here, depending on the level of noise, we want to find the amplitudes of signaling inputs with which an optimal decoder can make the best possible discrimination.

The Fisher information is defined as:

38 $$\begin{array}{@{}rcl@{}} \mathcal{I}_{\text{FI}}(A) = E \left[ \left( \frac{\partial \log f(\tau)} {\partial A} \right)^{2} \right]={\int}_{0}^{\infty} \frac{(\partial f(\tau)/\partial A)^{2}} {f(\tau) }\,d\tau, \end{array} $$

where the expectation is performed by *f*(*τ*)itself (see Eq. (34)). We assume the exponential decaying as signaling input’s functionality (i.e. Δ*I*(*t*,0) ∝ exp(−*t*/*τ*_s_), see Eq. (46) for details).

Figure 6a top, shows the Fisher information as a function of two scaled variables: amplitude, *A*/(*τ*_m_*V*_*𝜃*_), and noise level, $D / (\tau _{\mathrm {m}}V_{\theta }^{2})$. The dashed black lines locate the points of local maximums. Given the high noise level, $D/(\tau _{\mathrm {m}}V_{\theta }^{2})>0.088$, the Fisher information is maximized at a certain amplitude. The single maximum, however, splits into two maximums as the noise decreases. Figure 6a bottom, depicts the same $\mathcal {I}_{\text {FI}}(A)$ as a function of signal’s amplitude, for certain values of the noise level. There are two distinct maxima in the dark-red curve, $D/(\tau _{\mathrm {m}}V_{\theta }^{2})\simeq 0.01$. The two peaks, however, go down and approach each other as the noise level is increased; they finally merge into *one peak* for $D/(\tau _{\mathrm {m}}V_{\theta }^{2})\gtrsim 0.09$, the blue and dark-blue curves.

The mentioned two maximums which appear in low noise regime show noise plays a major role in optimal decoding. Despite the high noise level, the best discrimination would happen for two kinds of input strength, in the low noise level. The crucial role of noise is also studied in the context of mutual information, where the maximally informative solutions for neural population splits into two, as noise level decreases (Kastner et al. 2015). Figure 6a shows two branches for the maximum of the Fisher information. The left side branch indicates that the maximizing amplitude diminishes as noise decreases. A similar behavior has been seen using an extension of the *perfect integrate and fire* model (Levakova et al. 2016); however, they observed a single but not two maximums. Therefore, the existence of the second maximum, as a result of strong signaling input, is less expected and needs more exploration. Here, we suggest a hand-waving explanation, which intuitively explains the existence of the second peak for *strong amplitudes* in low noise levels.

The Fisher information (Eq. (38)) has an integral over *τ*; we may expect that the maximization of its integrand versus *A*, for certain domains of *τ*, results in the maximization of the whole $\mathcal {I}_{FI}(A)$. The integrand, also, is a fraction with *∂f*(*τ*)/*∂A* in its nominator, and *f*(*τ*)in its denominator. Figure 6c shows in the *logarithmic scale*, how *f*(*τ*) modifies as *A* increases. For large signal’s amplitude, as *A*/(*τ*_m_*V*_*𝜃*_) varies from 0.7 to 1, we see no significant change of *f*(*τ*)for 0 ≤ *τ* ≲ 2.5*τ*_s_ domain. On the contrary, for $\tau \gtrsim 2.5 \tau _{\mathrm {s}}$, we see a significant decrease in *f*(*τ*); increasing *A*/(*τ*_m_*V*_*𝜃*_)by a constant step of 0.1, always results in a significant downward shift of *f*(*τ*). The shift in the last step (*A*/(*τ*_m_*V*_*𝜃*_) : 0.9 → 1) seems almost twice as large as the shift in its previous step, in logarithmic scale. This picture suggests that as *A*/(*τ*_m_*V*_*𝜃*_) → 1, *f*(*τ*) drastically decreases, whereas *∂f*(*τ*)/*∂A* remains finite. This results in the growth of the integrand and hence the integral for the $\tau \gtrsim 2.5 \tau _{\mathrm {s}}$ domain.

Going back to the scenario of voltage trajectories, we can further understand this trend. A large transient signaling input boosts all trajectories with an upward shift of *A*/*τ*_m_. Consequently, those trajectories which are closer to the threshold than *A*/*τ*_m_, upon signal arrival, will fire immediately. What remains to fire afterward would be the trajectories which were below *V*_*𝜃*_ − *A*/*τ*_m_, upon signal occurrence. This picture lets us introduce two distinct sources for *f*(*τ*), during and after signal arrival. $F_{\text {during}}(A)={\int }_{V_{\theta }-A/\tau _{\mathrm {m}}}^{V_{\theta }} P_{\mathrm {s}}(V_{0})dV_{0}$ measures the portion of those trajectories which fire during signal arrival (e.g. 0 < *τ* < 2.5*τ*_s_), whereas $F_{\text {after}}(A)={\int }_{-\infty }^{V_{\theta }-A/\tau _{\mathrm {m}}} P_{\mathrm {s}}(V_{0})dV_{0}$ measures the portion which will reach the threshold after the signal arrival. For the case of exponentially decaying input, it roughly implies that:

39 $$\begin{array}{@{}rcl@{}} F_{\text{after}}(A)= {\int}_{-\infty}^{V_{\theta}-A/\tau_{\mathrm{m}}} P_{\mathrm{s}}(V_{0})\,dV_{0} \sim {\int}_{2.5 \tau_{\mathrm{s}}}^{\infty} f(\tau) d\tau. \end{array} $$

Equation (39) does not fully determine how *f*(*τ*)behaves after signal arrival; but it provides us a hint on how *f*(*τ*)varies with *A*. However, Fig. 6c helps us to go one step further; it shows a linear tail for *f*(*τ*) in *logarithmic scale*, *f*(*τ*) ∝ exp(−*αt*); all tails show almost the same slope: $\alpha \sim \tau _{\mathrm {m}}^{-1}$. This decouples the dependence of *f*(*τ*) on *A*, from its temporal dependence: *f*(*τ*) ∝ *F*_after_(*A*) × exp(−*αt*). Consequently, we would be able to estimate *∂f*(*τ*)/*∂A* which reads (*∂F*_after_(*A*)/*∂A*) × exp(−*αt*). The *∂F*_after_(*A*)/*∂A* simply is − (1/*τ*_m_)*P*_s_(*V*_*𝜃*_ − *A*/*τ*_m_). These points clarify how the integrand in Eq. (38) varies with *A*, in the 2.5*τ*_s_ < *τ* domain. The integrand and the whole integral would behave like:

40 $$\begin{array}{@{}rcl@{}} {\int}_{2.5 \tau_{\mathrm{s}}}^{\infty} \frac{(\partial f(\tau)/\partial A)^{2}} {f(\tau) }\,d\tau \propto \frac{P_{\mathrm{s}}(V_{\theta}-A/\tau_{\mathrm{m}})^{2}}{{\int}_{-\infty}^{V_{\theta}-A/\tau_{\mathrm{m}}} P_{\mathrm{s}}(V_{0})\,dV_{0}}. \end{array} $$

The right side of Eq. (40) has a simple geometrical interpretation. Consider the green curve in Fig. 6b (*D*/(*τ*_m_*V𝜃*2) = 0.2), the denominator of Eq. (40) equals the hatched area below the curve, while its nominator is simply the square of the height of that curve, *P*_s_(*V* ). As *A*/*τ*_m_ → *V*_*𝜃*_, the height of the curve remains finite (i.e. *P*_s_(0)), whereas the hatched area decreases to ${\int }_{-\infty }^{0} P_{\mathrm {s}}(V_{0})\,dV_{0}$.

We compare how the curve changes as noise decreases from green to red curve (Fig. 6b), $D/(\tau _{\mathrm {m}}V_{\theta }^{2})\,= 0.2\rightarrow 0.04$. There is almost a ratio of 2 between the heights of the curves at *V* = 0; this means that the nominator decreases by a factor of 1/2^2^. However, the hatched area is decreasing by a factor larger than 4. This means that the right side of Eq. (40) enlarges as noise level decreases. This trend is much stronger as noise level further decreases to $D/(\tau _{\mathrm {m}}V_{\theta }^{2})\,= 0.004$; the height is decreased by a factor of 0.63 whereas the enclosed area reduces to something hardly recognizable. In fact, we can show that the right side of Eq. (40) diverges like $(\tau _{\mathrm {m}}V_{\theta }^{2}/D)/\ln {(\tau _{\mathrm {m}}V_{\theta }^{2}/D)}$, as the noise level approaches to zero. So the integral of the Fisher information on the *after signal arrival domain* should diverge if both *A*/*τ*_m_ → *V*_*𝜃*_and $D/(\tau _{\mathrm {m}}V_{\theta }^{2}) \rightarrow 0$. This intuitive picture explains why the second peak arises for large amplitudes in low diffusion regime.

Figure 6a shows for weak amplitudes (0.02 ≤ *A*/(*τ*_m_*V*_*𝜃*_) < 0.1), when the level of noise increases, the Fisher information decreases monotonically. The same effect is observed when *A*/(*τ*_m_*V*_*𝜃*_) ≃ 1. But for the rest amount of amplitudes, the Fisher information is a non-monotonic function of noise level (see the dashed lines in Fig. 6a which show the maximum of the function); there is a certain level of the noise that maximizes the Fisher information (stochastic resonance) (Bulsara et al. 1991).

Finally, we can associate the two scaled diffusion and amplitude parameters with measurements from neural data. The diffusion coefficient relates to the variance of the noise distribution as *D* = *σ*^2^*τ*_m_/2. So the scaled diffusion coefficient would read $D/(\tau _{\mathrm {m}}V_{\theta }^{2})=\sigma ^{2}/ (2V_{\theta }^{2})$. If the variance of the noise distribution is known which is different for *in vivo* and *in vitro* neurons, the scaled diffusion parameter in Fig. 6a can be found. The scaled amplitude (*A*/(*τ*_m_*V*_*𝜃*_)) also shows the measure of excitatory postsynaptic potential (EPSP) which is available in different experimental studies (Shadlen and Newsome 1994; Song et al. 2005; Lefort et al. 2009; Cossell et al. 2015).

## Discussion

In this study, we analytically derived the statistical input-output relation of a LIF neuron receiving transient signaling input on top of noisy balanced inputs. We developed a first-passage time density of the neuron when it receives the signal at the threshold regime. We examined a simple square input signal, and then extended it to more physiologically plausible signaling inputs. Our prediction matches well with simulation study, which shows the applicability of our model for more realistic signaling input shapes. The first-passage time density is a function of the scaled diffusion coefficient and the scaled amplitude of signaling input. It also depends on the arrival time of the signaling input elapsed from the last postsynaptic spike. We also extended our analysis and made it independent from the knowledge of the last postsynaptic spikes by marginalizing over all possible last postsynaptic spikes with respect to input arrival time. Based on the analytic expression for the first-spiking density after input arrival, we examined the Fisher information with respect to signaling input’s amplitude (efficacy). The result reveals that for each level of the noise, there are specific amplitudes of signaling inputs at which the decoding can be done most accurately.

Here, we investigate the LIF neuron model (Stein 1965). Although lots of studies have shown that some extended models such as adaptive exponential integrate-and-fire model (aEIF) better explains the neural properties (Izhikevich 2004; Ostojic and Brunel 2011), the LIF neuron model can capture the properties of the cortical pyramidal neurons (Rauch et al. 2003; La Camera et al. 2004; Jolivet et al. 2008); it is still the widely studied neuron model because of its simplicity to be driven analytically (Burkitt 2006a, 2006b).

Previous studies on the *LIF neuron model* already attempted to find an analytical solution for the first-passage time density or firing rate in the presence of signaling input in the noisy balanced environment. The effect of small oscillatory input on the first-passage time problem is studied by Bulsara *et.al.* using image method to solve the Fokker-Planck equation (Bulsara et al. 1996). The linear response of LIF neuron to oscillatory input and the change of firing rate starting from stationary distribution is also investigated analytically in a Fokker-Planck formalism by some studies (Brunel and Hakim 1999; Brunel et al. 2001; Lindner and Schimansky-Geier 2001). Richardson and Swarbrick provided analytical result for firing rate modulation up to linear order receiving excitatory and inhibitory synaptic jumps drawn from the exponential distribution (Richardson and Swarbrick 2010). In addition, there are studies that investigated the effect of transient input current (Herrmann and Gerstner 2001; Helias et al. 2010). Hermann and Gerstner showed how the post-stimulus time histogram is changed by the transient input signal on top of noise using the escape rate model and hazard function. They provided a numerical solution for the full model but the analytical result is achievable up to the first order. Moreover, Helias and coworkers (Helias et al. 2010) found the effect of the delta input kick in the Fokker-Planck equation on the firing rate of postsynaptic neuron up to linear order, assuming steady state distribution for finite synaptic amplitudes prior to input arrival.

Here, we analytically solved the first-passage time density for the LIF neuron receiving transient signaling input with arbitrary amplitude and shape on top of Gaussian background. However, it is obtained under the assumption that the mean input drive equals or at least is near to the threshold potential (see Appendix I). Further, it was assumed that the synaptic time constant is considerably smaller than the membrane time constant. However, within these limitations, we can analytically achieve various features of the LIF neuron’s spiking density. Our framework and approach at first is conditioned on the knowledge of last postsynaptic spike; this framework is useful in some experimental studies which the last postsynaptic spike is assumed (Froemke and Dan 2002; Wang et al. 2005; Blot et al. 2016) or the neuron may learn to time sequential responses (Johansson et al. 2016; Jirenhed et al. 2017). Moreover, we show by using the backward recurrence time distribution, the first-spiking density after input arrival is achieved, which does not depend on the last postsynaptic spike (see Eq. (34)); it can be used for experiments that the knowledge of last postsynaptic spike is not considered and just the time of input arrival is assumed as a reference signal for readout by downstream neurons (Van Rullen et al. 2005; Panzeri et al. 2014). We also show that the result of using backward recurrence time for the marginalization of the spiking density, in Eq. (34), can be achieved by another approach (see Eq. (37)) assuming a stationary distribution at the time of input arrival and the use of Green’s function from Eq. (6). While Eq. (34) has just one integral over time and computationally is faster, Eq. (37) gives us intuition about the spiking density after input arrival, which was used in the Fisher information part to describe the second observed maximum in low noise regime.

In this study, we model the background synaptic activity as a white *Gaussian noise*. This would be a too simplified view on the background activity. For example, background synaptic inputs to neurons may be described as shot noise (Richardson and Swarbrick 2010; Helias et al. 2013) and is synaptically filtered (Brunel and Sergi 1998; Moreno-Bote and Parga 2010). It can also be modeled as temporally correlated noise (colored noise) because of long lasting time scale of NMDA and GABA_*B*_generated currents (Lerchner et al. 2006; Dummer et al. 2014; Ostojic 2014). However, the white Gaussian noise still can be a reasonable assumption for short lasting currents generated by AMPA and GABA_*A*_ receptors (Destexhe et al. 1998). In fact, in the limit of *τ*_*s*_ ≪ *τ*_m_, and with small numerous background synaptic amplitudes, the background activity is approximated by *white Gaussian noise*. Moreover, experimental evidence reported temporally uncorrelated noise for an animal engaged in a task (Poulet and Petersen 2008; Tan et al. 2014). The Gaussian noise, however, changes to correlated noise for an anesthetized animal, or when the animal is in its quiet wakefulness. Hence it would be important to extend the current formalism to non-Gaussian situations, as well.

Moreover, our solution works for the neurons at the threshold regime, at which the membrane potential of the neurons are very close to the threshold. At this regime, even small fluctuation brings the membrane potential above the threshold voltage, which makes the neuron to fire. Recent experimental study (Tan et al. 2014) shows that this scenario works when stimulus is presented to the monkey. To check how the solution changes when the value of the mean input deviates from the threshold voltage (sub/supra-threshold), we also use scaling approach and compare it with simulation study (Appendix I). The result shows, by scaling relation that we introduced, we can go beyond threshold spiking density to near sub/supra-threshold densities and extend our solution to more plausible cases of sub/supra-threshold regimes.

Finally, we calculate the Fisher information based on spike timing rather than rate or spike count (Rieke et al. 1999; van Vreeswijk 2001; Toyoizumi et al. 2006). Precise spike timing of postsynaptic neuron relative to signaling input arrival has information that may be lost or decreased in spike count methods that, spikes (responses) are summed over long time windows (Panzeri et al. 2010). The Fisher information as one application of the first-spiking density is investigated here; we find the specific amplitude of signaling inputs that can be distinguished most accurately by downstream neurons. To maximize the accuracy of decoding in low noise level, neurons have two choices for their synaptic strength, one in weak and the other in strong amplitude. But for higher levels of the noise, there is one strong amplitude which maximizes the FI; the achieved maximum is robust to a mere change of the amplitude (Fig. 6a Bottom, blue color). This effect may have some advantage in neural decoding and learning; strong amplitudes which might be the result of causal Hebbian learning (Dan and Poo 2004) can be discriminated most accurately by downstream neurons, even in low noise regime. We also revisit the stochastic resonance (Douglass et al. 1993; McDonnell and Ward 2011; Teramae et al. 2012; Ikegaya et al. 2013; Levakova et al. 2016); the Fisher information does not behave monotonically with the noise level, instead, there exists a wide range of signaling input’s amplitude for which the Fisher information is maximized in a certain and finite value of the noise (Fig. 6a).

The input-output relation of a single neuron embedded in a network is the building block of the neural activity underlying learning, cognition and behavior. Since strong synaptic inputs are pervasive in the neural system (Song et al. 2005; Lefort et al. 2009; Ikegaya et al. 2013; Buzsáki and Mizuseki 2014; Cossell et al. 2015), the analytic solution that can deal with the effect of strong transient signaling inputs can widely be used in predicting network’s complex activity (Herz et al. 2006). Given that the LIF model describes spike timing with adequate accuracy, the analytic solution presented here is expected to facilitate theoretical investigation of information processing in the neural systems.

## Figures and Tables

**Fig. 7:**
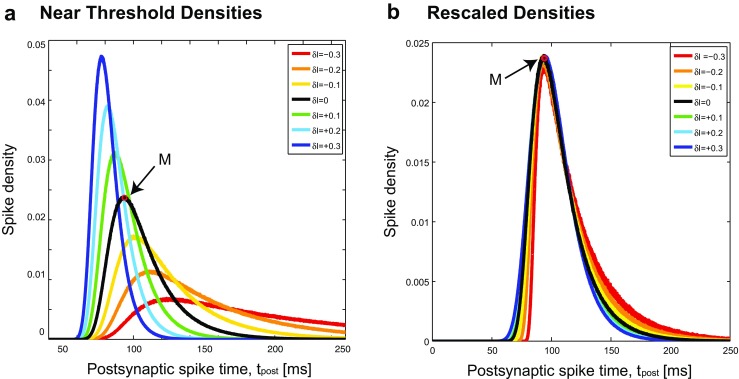
Rescaling the First-passage time densities of different supra-threshold and sub-threshold currents near the threshold. **a** First-passage time densities for different *δI* from simulation study. The minus sign for *δI* shows sub-threshold currents while the plus sign shows supra-threshold currents. As we go further away from the threshold regime to supra-threshold currents, the spiking density gets sharper and more symmetric but in sub-threshold currents, by going further from threshold current, the density would be more asymmetric with a long tale and the peak shifts to the right side. The black curve is the density at the threshold regime (*δI* = 0). The red dot (*M*) shows the maximum of the density where is the reference point for the scaling of the densities. **b** Collapse of all the rescaled supra-threshold and sub-threshold spiking densities on the analytic spiking density at the threshold. Each individual density is rescaled by transferring its $t_{\max }$ and *r* by Eq. (41). Each curve is achieved by simulation study for 10^11^runs to have a smooth graph. The parameters used are *τ*_m_ = 20*ms*, *V*_*𝜃*_ = 20*mV*, *D* = 0.74 mV^2^msand *δI* = − 0.3,− 0.2,− 0.1,0,0.1,0.2,0.3. Simulation time step is 0.05*ms*

**Fig. 8:**
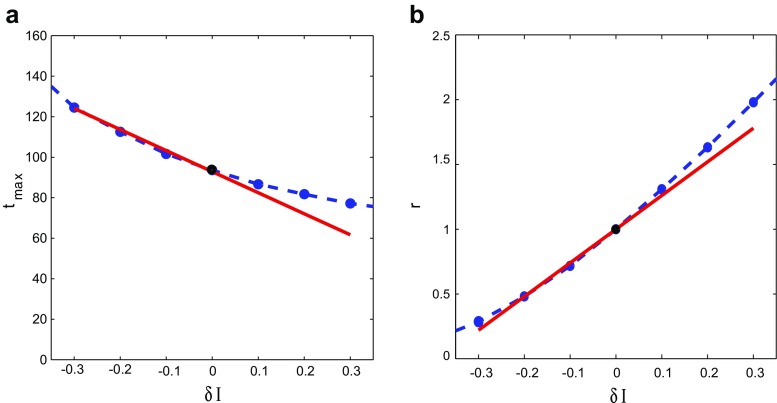
Comparing simulation with our analytical scaling relations (Eqs. (43) and (44)). **a**$t_{\max }$ as a function of the deviation of the drive current from threshold (*δI*) is shown for simulation study (blue dots and blue dashed line) comparing with analytical scaling relation (red line, Eq. (44)). It shows good agreement between simulation and analytical study especially for sub-threshold regimes. **b** The ratio (*r*) between $J_{\max }(\delta I)$ and the maximum of the threshold curve as a function of *δI* for both simulation and analytical study. The analytical result for *r* (Eq. (43), red line) also agrees well with simulation study (blue dots and blue dashed line), especially for sub-threshold currents. The black dot in each graph shows the result of the threshold regime (*δI* = 0). The parameters used are *τ*_m_ = 20 ms, *V*_*𝜃*_ = 20 mV, *D* = 0.74 mV^2^msand *δI* = − 0.3,− 0.2,− 0.1,0,0.1,0.2,0.3. Simulation time step is 0.05*ms* and each point is achieved by 10^11^runs

**Fig. 9:**
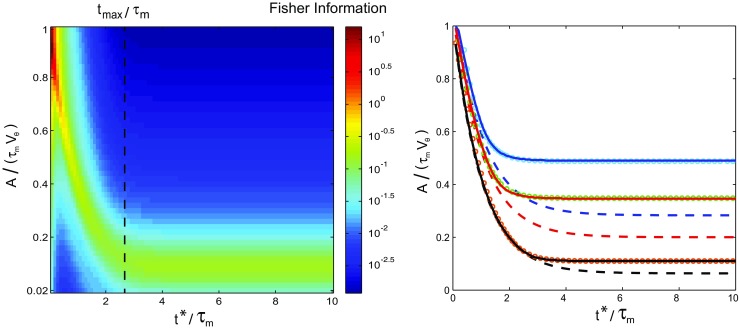
Fisher information with respect to the amplitude of signaling input when the decoder has access to the last postsynaptic spike. Left: Fisher information in logarithm scale as a function of the scaled amplitude, *A*/(*τ*_m_*V*_*𝜃*_), and the scaled presynaptic time, *t*^∗^/*τ*_m_. The signaling input is the exponential decaying function(Eq. (46), *τ*_*s*_ = 1 ms) while the diffusion coefficient is fixed here, *D*/(*τ*_m_*V𝜃*2) = 0.004. The Fisher information is maximized when the signaling input arrives immediately with strong amplitude *A*/(*τ*_m_*V*_*𝜃*_) = 0.92which generates the postsynaptic neuron to fire immediately with minimum contribution from the noise. Dashed black line shows the time when $t^{*}=t_{\max }$. In the $t^{*} <t_{\max }$ region, the Fisher information decreases monotonically. The later the signaling input arrives, we would need higher amplitude to maximize information; this maximum monotonically decreases with the arrival time. For $t^{*} > t_{\max }$, the maximizing amplitude and Fisher information are constants and independent of *t*^∗^. Right: the maximizing scaled amplitude, *A*/(*τ*_m_*V*_*𝜃*_)as a function of normalized arrival time (*t*^∗^/*τ*_m_) for different diffusion coefficients(*D*/(*τ*_m_*V𝜃*2) = 0.004,0.04,0.08corresponding to black, red and blue). The circles show the maximum of Fisher information from Eq. (38). The dashed lines show Eq. (83) which has a good match for $t^{*} < t_{\max }$, but deviates for $t^{*}> t_{\max }$. The Solid lines show *∂*^2^*P*_0_(*V*,*t*)/*∂V*^2^ = 0(see Eq. (7)) which has excellent compatibility with the circles

## Appendix I: Spiking density near the threshold regime

The suggested image method solution is valid exclusively for the threshold regime, when the drive current equals the threshold voltage (i.e. $\bar {I}=V_{\theta }$). In the ubiquitous *near threshold* regime, however, the drive current is close but not exactly equal to the threshold voltage, $\bar {I}=V_{\theta }+\delta I $. Thus we extend our formalism beyond, to address such a situation. Figure 7a depicts the first-passage time density for various values of $\bar {I}$. In Fig. 7b, we have *rescaled* all these curves; all of them collapse with the analytic solution at $\bar {I}=V_{\theta }$.

The scaling is simply based on transferring the maximum point of either of the curves to point **M**, the maximum of the threshold curve (red dot in Fig. 7a). We locate *t*_max_(*δ**I*)and *J*_max_(*δ**I*), which are the *occurrence time* and the *value of spiking density*, at the maximum point of the curve with $\bar {I}=V_{\theta }+\delta I$. We find the ratio between *J*_max_(*δ**I*) and the maximum of the threshold curve: *r* = *J*_max_(*δ**I*)/*J*_max_(0). Each curve in Fig. 7a transfers to its corresponding curve in Fig. 7b according to:

41 $$\begin{array}{@{}rcl@{}} \left( \begin{array}{c} t \\ J_{\delta I} \end{array} \right) \rightarrow \left( \begin{array}{c} t_{\max}(0)+r\times (t-t_{\max}(\delta I))\\ (1/r)\times J_{\delta I} \end{array} \right) \end{array} $$

The second row, states that the height of the curve is tuned; so the height of its maximum would be equal to the maximum height of the threshold solution. Similarly, the first row tunes the occurrence time of the maximum. Moreover, it shows that the width of the curve is scaled reversely, compared to its height. This guarantees that the surface below the curve does not change, but remains normalized to one. After scaling, the curves collapse to the analytic solution. Therefore we can reversely use the analytic solution to find the spiking density for the near threshold curves:

42 $$\begin{array}{@{}rcl@{}} J_{\delta I}(t)= r \times J_{\text{analytic}}(t_{\max}(0)+r\times (t-t_{\max}(\delta I))), \end{array} $$

So this scaling shows that if we know the analytic expression of *r* and *t*_max_(*δ**I*), we can easily find the spiking density for the near threshold curves from analytic threshold density. If $\tau _{\mathrm {m}} V_{\theta }^{2} \gg D $ and *δ**I* ≪ *V*_*𝜃*_, We can find the analytic expressions for *r* and *t*_max_(*δ**I*) up to first order with respect to *δ**I*:

43 $$\begin{array}{@{}rcl@{}} r = 1+\frac{1}{2}\sqrt{\frac{\tau_{\mathrm{m}} V_{\theta}^{2}}{D}} \frac{\delta I}{V_{\theta}}, \end{array} $$

and

44 $$\begin{array}{@{}rcl@{}} t_{\max}(\delta I)= \frac{1}{2}\tau_{\mathrm{m}} \ln{\left( \frac{\tau_{\mathrm{m}} V_{\theta}^{2}}{D}\right)}-\tau_{\mathrm{m}} \sqrt{\frac{\tau_{\mathrm{m}} V_{\theta}^{2}}{D}} \frac{\delta I}{V_{\theta}}. \end{array} $$

Figure 8 shows the linear analytic expressions (Eqs. (43) and (44)) have good match with simulation data especially at the sub-threshold regimes.

The idea behind these analytic relations (Eqs. (43) and (44)) is as follows. We put the threshold aside for a moment. When we have no noise, the voltage trajectory in Eq. (1) is easily driven by $V_{\det }=\bar {I}(1-\exp {[-t/\tau _{\mathrm {m}}]})$. In the presence of just white noise, the voltage of LIF is given by $\delta V_{\text {noise}}=\sqrt {D(1-\exp {[-t/\tau _{\mathrm {m}}]})/\tau _{\mathrm {m}}}$. We imitate some ideas come from the Brownian motion problems, and put forward an open conjecture. Fortunately, the conjecture happens to work finely. To address when the trajectories, on average, reach the threshold, we consider the time when *V*_det_ + *δ**V*_noise_ = *V*_*𝜃*_:

45 $$\begin{array}{@{}rcl@{}} &&\bar{I}(1-\exp{[-t_{\max}/\tau_{\mathrm{m}}]})\\ &&\qquad \quad \quad +\sqrt{D(1-\exp{[-t_{\max}/\tau_{\mathrm{m}}]})/\tau_{\mathrm{m}}}=V_{\theta}, \end{array} $$

where $\bar {I}=V_{\theta }+\delta I$. Expanding Eq. (45) up to the first order with respect to *δ**I* and assuming $\frac {\tau _{\mathrm {m}} V_{\theta }^{2}}{D} \gg 1$, we reach to Eq. (44). For finding analytical expression for *r*, we put the free Green function (see Eq. (5)) in first-passage time density *J* (see Eq. (8)) while $\bar {I}=V_{\theta }+\delta I$. If we expand the expression up to first order with respect to *δ**I*, for $\frac {\tau _{\mathrm {m}} V_{\theta }^{2}}{D} \gg 1$, the result leads to Eq. (43).

## Appendix II: Exponential decaying function as signaling input

Here, we extend our method to more physiologically plausible signaling input case, exponential decaying function. We assume that the signaling input has the form of:

46 $$\begin{array}{@{}rcl@{}} {\Delta} I(t, t^{*})=A \times \left\{\begin{array}{l} 0 {\quad\quad\quad\qquad\qquad\qquad\ \ } t < t^{*},\\ {\frac{1}{\tau_{s}}}\, {\exp\left( -\frac{t-t^{*}}{\tau_{\mathrm{s}}}\right)} {\qquad\quad}\,\,\,\, t^{*} \leq t. \end{array}\right. \end{array} $$

where *τ*_*s*_ is the synaptic time constant and *τ*_*s*_ ≪ *τ*_m_. Based on the defined signaling input current, to get the result of the first order, one should solve Eq. (14). To take the time integral of Eq. (15), we use the same reasoning with the assumption *τ*_*s*_ << *τ*_m_ which simplifies the result to Eq. (16) where *f*_1_(*t*,*t*^∗^)for *t* ≥ *t*^∗^is:

47 $$\begin{array}{@{}rcl@{}} f_{1}(t,t^{*})=-\left( 1-e^{-\frac{t-t^{*}}{\tau_{s}}}\right) \end{array} $$

and *f*_1_(*t*,*t*^∗^) = 0 for *t* < *t*^∗^. for second and higher order terms of perturbations, *n* ≥ 2, we have the same result as before (see Eq. (18)) but with different *f*_*n*_(*t*,*t*^∗^)(see Appendix II):

48 $$\begin{array}{@{}rcl@{}} f_{n}(t,t^{*})\,=\,(-1)^{n}\left( \frac{1}{n!}-\frac{e^{-\frac{t-t^{*}}{\tau_{s}}}}{1!(n\,-\,1)!}\,+\,\frac{e^{-2 \frac{t-t^{*}}{\tau_{s}}}}{2!(n-2)!}-...\right) \end{array} $$

To find the final result, we should add all *n* terms as in Eq. (12) and obtain Δ*P*(*V*,*t*). For *t* > *t*^∗^, it reads:

49 $$\begin{array}{@{}rcl@{}} {\Delta} P(V,t) = {\int}_{V_{0}=-\infty}^{V_{\theta}} G(V,t;V_{0},t^{*})Q(V_{0},t,t^{*}) \,d V_{0}, \end{array} $$

where *Q*(*V*_0_,*t*,*t*^∗^)is:

50 $$\begin{array}{@{}rcl@{}} Q(V_{0},t,t^{*})\,=\,{\Sigma}_{n = 1}^{n=\infty} f_{n}(t,t^{*}) \left( \frac{A}{\tau_{\mathrm{m}}}\right)^{n} \frac{\partial^{n}}{\partial {V_{0}^{n}}}P_{0}(V_{0} ,t^{*}). \end{array} $$

If we put *f*_*n*_(*t*,*t*^∗^) from Eq. (48) in Eq. (50), the first term $(-1)^{n}\frac {1}{n!}$ in Eq. (50), simplifies to:

51 $$\begin{array}{@{}rcl@{}} {\Sigma}_{n = 1}^{n=\infty}\frac{(-\frac{A}{\tau_{\mathrm{m}}})^{n}}{n!}\frac{\partial^{n}}{\partial {V_{0}^{n}}}P_{0}(V_{0} ,t^{*})&\,=\,&P_{0}\left( V_{0}-\frac{A}{\tau_{\mathrm{m}}},t^{*}\right)\\ &&-P_{0}(V_{0},t^{*}). \end{array} $$

The second term of *f*_*n*_(*t*,*t*^∗^), $-(-1)^{n}\frac {e^{-\frac {t-t^{*}}{\tau _{s}}}}{1!(n-1)!}$, in Eq. (50) leads to:

52 $$\begin{array}{@{}rcl@{}} \frac{A }{\tau_{\mathrm{m}}}e^{-\frac{t-t^{*}}{\tau_{s}}}{\Sigma}_{n = 1}^{n=\infty}\frac{(-\frac{A}{\tau_{\mathrm{m}}})^{n-1}}{(n-1)!}\frac{\partial^{n-1}}{\partial V_{0}^{n-1}}\left( \frac{\partial}{\partial V_{0}}P_{0}(V_{0},t^{*})\right)\\ = \frac{A}{\tau_{\mathrm{m}}}e^{-\frac{t-t^{*}}{\tau_{s}}}\frac{\partial}{\partial V_{0}}P_{0}\left( V_{0}-\frac{A}{\tau_{\mathrm{m}}},t^{*}\right). \end{array} $$

Simply we can calculate all the other terms in Eq. (50) and add them together. Then, *Q*(*V*_0_,*t*,*t*^∗^) would be:

53 $$\begin{array}{@{}rcl@{}} Q(V_{0},t,t^{*})&=&-P_{0}(V_{0},t^{*})\\ &&+P_{0}\left( V_{0}-\frac{A}{\tau_{\mathrm{m}}}\left( 1-e^{-\frac{t-t^{*}}{\tau_{s}}}\right), t^{*}\right). \end{array} $$

Finally the result of *P*(*V*,*t*) = *P*_0_(*V*,*t*) + Δ*P*(*V*,*t*), by inserting Eq. (53) into Eq. (49), simplifies as:

54 $$\begin{array}{@{}rcl@{}} P(V,t)&=&{\int}_{V_{0}=-\infty}^{V_{\theta}} G(V,t;V_{0},t^{*}) \\ &&\times P_{0}\left( V_{0}- {\frac{A}{\tau_{\mathrm{m}}}}\left( 1-e^{-\frac{t-t^{*}}{\tau_{s}}}\right)\,, t^{*}\right)\,d V_{0}. \end{array} $$

The whole signature of the input current is gathered in a single term, ${\frac {A}{\tau _{\mathrm {m}}}}(1-e^{-\frac {t-t^{*}}{\tau _{s}}})$; this term, however, is $(1/\tau _{\mathrm {m}}){\times {\int }_{0}^{t}} {\Delta } I(s, t^{*}) ds$. Consequently, the result could also be written as:

55 $$\begin{array}{@{}rcl@{}} P(V,t)&=&{\int}_{V_{0}=-\infty}^{V_{\theta}} G(V,t;V_{0},t^{*}) \\ &&\times P_{0}\left( V_{0}-\!{{\int}_{0}^{t}} \frac{\Delta I(s, t^{*})}{\tau_{\mathrm{m}}} ds\,, t^{*}\right)\,d V_{0}. \end{array} $$

The first-passage time density is also simply attainable by inserting Eq. (55) into Eq. (25):

56 $$\begin{array}{@{}rcl@{}} J(V_{\theta},t)&=&-\frac{D}{\tau_{\mathrm{m}}^{2}}{\int}_{V_{0}=-\infty}^{V_{\theta}} \tilde{G}(V_{\theta},t;V_{0},t^{*})\\ && \times P_{0}\left( V_{0}-\!{{\int}_{0}^{t}} \frac{\Delta I(s, t^{*})}{\tau_{\mathrm{m}}} ds\,, t^{*}\right)\,d V_{0}, \end{array} $$

where $\tilde {G}(V_{\theta },t;V_{0},t^{*})=(\partial G(V,t;V_{0},t^{*})/\partial V)|_{V=V_{\theta }}$.

### Appendix II-a

For an exponential decaying signaling input, Eq. (46), the first order term (see Eq. (15)) can be simplified by taking the integral of Δ*I*(*t*,*t*^∗^) over *t*:

57 $$\begin{array}{@{}rcl@{}} -{{\int}_{0}^{t}}\frac {\Delta I(t,t^{*})}{\tau_{\mathrm{m}}} dt = -\frac{A}{\tau_{\mathrm{m}}} \left( 1-e^{-\frac{t-t^{*}}{\tau_{s}}}\right) \end{array} $$

which leads to Eq. (16) where *f*_1_(*t*,*t*^∗^)is:

58 $$\begin{array}{@{}rcl@{}} f_{1}(t,t^{*})=-\left( 1-e^{-\frac{t-t^{*}}{\tau_{s}}}\right) \end{array} $$

The integral of current Δ*I*(*t*,*t*^∗^)for the second term of perturbation is:

59 $$\begin{array}{@{}rcl@{}} {{\int}_{0}^{t}}\left( \frac{\Delta I(t,t^{*})}{\tau_{\mathrm{m}}}\right)^{2} dt \,=\, \left( \frac{A}{\tau_{\mathrm{m}}}\right)^{2\!} \left( \frac{1}{2}-e^{-\frac{t-t^{*}}{\tau_{s}}}\,+\,\frac{1}{2}e^{-2\frac{t-t^{*}}{\tau_{s}}}\right) \end{array} $$

which leads to Eq. (18) when *n* = 2with:

60 $$\begin{array}{@{}rcl@{}} f_{2}(t,t^{*})=\frac{1}{2!}-e^{-\frac{t-t^{*}}{\tau_{s}}}+\frac{1}{2!}e^{-2\frac{t-t^{*}}{\tau_{s}}} \end{array} $$

We can also go further and find the integral of current for third and fourth order of perturbation. The integral of third order is:

61 $$\begin{array}{@{}rcl@{}} {\kern-1.5pt}-\!{{\int}_{0}^{t}}\!\!\left( \!\frac{\Delta{\kern-.5pt} I{\kern-.5pt}({\kern-.5pt}t{\kern-.5pt},{\kern-.5pt}t^{*}{\kern-.5pt})}{\tau_{\mathrm{m}}}\!\right)^{3} dt\! &=&\! \left( -\frac{A}{\tau_{\mathrm{m}}}\right)^{3} \\ &&\!\!\times\!\left( {\kern-.7pt}\frac{1}{6}{\kern-.5pt}-{\kern-.5pt}\frac{1}{2}e^{-\frac{t-t^{*}}{\tau_{s}}}{\kern-.7pt}+{\kern-.5pt}\frac{1}{2}e^{-2\frac{t-t^{*}}{\tau_{s}}}\right. \left.\!-\frac{1}{6}e^{-3\frac{t-t^{*}}{\tau_{s}}}\!\right),\\ \end{array} $$

where *f*_3_(*t*,*t*^∗^)is:

62 $$\begin{array}{@{}rcl@{}} f_{3}(t,t^{*})\,=\,-\left( \frac{1}{3!}\,-\,\frac{1}{2!}e^{-\frac{t-t^{*}}{\tau_{s}}}\,+\,\frac{1}{2!}e^{-2\frac{t-t^{*}}{\tau_{s}}}\,-\, \frac{1}{3!}e^{-3\frac{t-t^{*}}{\tau_{s}}}\right) \end{array} $$

For the fourth order we have:

63 $$\begin{array}{@{}rcl@{}} &&{}{{\int}_{0}^{t}}\left( \frac{\Delta I(t,t^{*})}{\tau_{\mathrm{m}}}\right)^{4} dt = \left( \frac{A}{\tau_{\mathrm{m}}}\right)^{4} \\ &&\times\left( \frac{1}{4!}-\frac{1}{3!}e^{-\frac{t-t^{*}}{\tau_{s}}}+\frac{1}{4}e^{-2\frac{t-t^{*}}{\tau_{s}}}-\frac{1}{3!} e^{-3\frac{t-t^{*}}{\tau_{s}}}+\frac{1}{4!}e^{-4\frac{t-t^{*}}{\tau_{s}}}\right). \end{array} $$

This also leads to Eq. (18) for *n* = 4with:

64 $$\begin{array}{@{}rcl@{}} f_{4}(t,t^{*})&=& \frac{1}{4!}-\frac{1}{3!}e^{-\frac{t-t^{*}}{\tau_{s}}}+\frac{1}{4}e^{-2 \frac{t-t^{*}}{\tau_{s}}}-\frac{1}{3!}e^{-3\frac{t-t^{*}}{\tau_{s}}}\\ &&+\frac{1}{4!}e^{-4\frac{t-t^{*}}{\tau_{s}}} \end{array} $$

Comparing Eqs. (58), (60), (62) and (64) together, *f*_*n*_(*t*,*t*^∗^)simply is:

65 $$\begin{array}{@{}rcl@{}} f_{n}(t,t^{*})&=&(-1)^{n}\left( \frac{1}{n!}-\frac{e^{-\frac{t-t^{*}}{\tau_{s}}}}{(n-1)!}+\frac{e^{-2\frac{t-t^{*}}{\tau_{s}}}}{2!(n-2)!}\right.\\ &&\left.-\frac{e^{-3\frac{t-t^{*}}{\tau_{s}}}}{3!(n-3)!}+...+(-1)^{n}\frac{e^{-n\frac{t-t^{*}}{\tau_{s}}}}{n!}\right) \end{array} $$

## Appendix III: Extending first-passage time density to multiple presynaptic spike times

If more than one presynaptic spike occurs after reset time, as far as the input’s spikes and shapes don’t interfere with each other, the first-passage time density is simply a superposition of the results. To make it clear, we assume a case that first presynaptic spike arrives at $t^{*}_{1}$ and the second pre spike arrives at $t^{*}_{2}$ (*t*2∗ > *t*1∗). The first-passage time density of postsynaptic neuron after $t^{*}_{1}$ is derived by Eqs. (24) and (25) but after $t^{*}_{2}$ is:

66 $$\begin{array}{@{}rcl@{}} J{\kern-.5pt}({\kern-.5pt}V_{\theta}{\kern-.5pt},{\kern-.5pt}t{\kern-.5pt})\! &=&\! -\frac{D}{\tau_{\mathrm{m}}^{2}}{\int}_{-\infty}^{V_{\theta}}d V_{1} {\int}_{-\infty}^{V_{\theta}}d V_{2} \tilde{G}(V_{\theta},t;V_{2},t^{*}_{2})\\ &&\times {G}\! \left( \! V_{2}{\kern-.5pt}-{\kern-.5pt}{\frac{A}{\tau_{\mathrm{m}}}},t^{*}_{2}{\kern-.5pt},{\kern-.5pt}V_{1}{\kern-.5pt},{\kern-.5pt}t^{*}_{1}{\kern-.5pt}\right)\! P_{0}\! \left( \!V_{1}\! -\! {\frac{A}{\tau_{\mathrm{m}}}}, t^{*}_{1}\right)\!, \end{array} $$

where $\tilde {G}(V_{\theta },t;V_{2},t^{*}_{2})=\frac {\partial G(V,t;V_{2},t^{*}_{2})}{\partial V}|_{V=V_{\theta }}$. The integration can be taken numerically by software.

## Appendix IV: Analytic expression for first-passage time density

Here we calculate the analytic expression for First-passage time density. By putting Eq. (29) and *P*_0_ from Eq. (7) in Eq. (28) and changing the variable (*V*_*𝜃*_ − *V*_0_ = *y*), the first-passage time density is:

67 $$\begin{array}{@{}rcl@{}} J(V_{\theta},t)&\,=\,&\gamma(t,t^{*}){\int}_{0}^{\infty}y dy ~e^{-\alpha(t-t^{*}) y^{2}/2}\\ && \times \left[e^{-\beta(t^{*})(y-S_{+}(t,t^{*}))^{2}/2}\,-\,e^{-\beta(t^{*})(y-S_{-}(t,t^{*}))^{2}/2}\right], \end{array} $$

where *γ*(*t*,*t*^∗^), *α*(*t* − *t*^∗^), *β*(*t*^∗^) and *S* ± (*t*,*t*^∗^)are:

68 $$\begin{array}{@{}rcl@{}} \gamma(t,t^{*})&=&\frac{1}{\pi D }\frac{r(t-t^{*})}{\sqrt{(1-r^{2}(t^{*}))(1-r^{2}(t-t^{*}))^{3}}}\\ \alpha(t-t^{*})&=&\frac{\tau_{\mathrm{m}}}{D}\frac{r^{2}(t-t^{*})}{1-r^{2}(t-t^{*})}\\ \beta(t^{*})&=&\frac{\tau_{\mathrm{m}}}{D}\frac{1}{1-r^{2}(t^{*})}\\ S_{\pm}(t,t^{*})&=&-\frac{1}{\tau_{\mathrm{m}}}{{\int}_{0}^{t}}dq{\Delta} I(q,t^{*}) \-\pm r(t^{*})V_{\theta}\\ r(t)&=&e^{\frac{-t}{\tau_{\mathrm{m}}}}. \end{array} $$

The exponential term in Eq. (67) for the first(or the second) term, *S*_±_ can be written in quadratic form:

69 $$\begin{array}{@{}rcl@{}} e^{-\alpha y^{2}/2}e^{-\beta(y-S)^{2}/2}=e^{-\frac{\alpha+\beta}{2}\left( y-\frac{\beta S}{\alpha+\beta}\right)^{2}} e^{-\frac{S^{2}}{2}\frac{\alpha \beta}{\alpha+\beta}}, \end{array} $$

where each of the two terms of integral in Eq. (67) is simplified to:

70 $$\begin{array}{@{}rcl@{}} e^{-\frac{S^{2}}{2}\frac{\alpha \beta}{\alpha+ \beta}}{\int}_{0}^{\infty} y e^{-\frac{\alpha+\beta}{2}\left( y-\frac{\beta S}{\alpha+\beta}\right)^{2}}dy, \end{array} $$

by changing the variable $y-\frac {\beta S}{\alpha +\beta }=u$, the integral over *y* in Eq. (70) can be taken:

71 $$\begin{array}{@{}rcl@{}} {\int}_{\frac{-\beta S}{\alpha+\beta}}^{\infty}u e^{-\frac{\alpha + \beta}{2}u^{2}}du +{\int}_{\frac{-\beta S}{\alpha+\beta}}^{\infty}\frac{\beta S}{\alpha+\beta}e^{-\frac{\alpha+\beta}{2}u^{2}}du, \end{array} $$

each of the integrals in Eq. (71) can be written as two terms; one integral from $-\frac {\beta S}{\alpha +\beta }$ to 0 and the other term from 0 to *∞*:

72 $$\begin{array}{@{}rcl@{}} {\int}_{-\frac{\beta S}{\alpha+\beta}}^{0}u e^{-\frac{\alpha + \beta}{2}u^{2}}du&+&{\int}_{0}^{\infty}u e^{-\frac{\alpha + \beta}{2}u^{2}}du\\ &+&{\int}_{-\frac{\beta S}{\alpha+\beta}}^{0}\frac{\beta S}{\alpha+\beta}e^{-\frac{\alpha+\beta}{2}u^{2}}du\\ &+&{\int}_{0}^{\infty}\frac{\beta S}{\alpha+\beta}e^{-\frac{\alpha+\beta}{2}u^{2}}du. \end{array} $$

The first, second and fourth integral in Eq. (72) can be taken analytically. So the final result of Eq. (70) is:

73 $$\begin{array}{@{}rcl@{}} e^{-\frac{S^{2}}{2}\frac{\alpha \beta}{\alpha+ \beta}}\left( \frac{1}{\alpha+\beta}e^{-\frac{\beta^{2} S^{2}}{2(\alpha+\beta)}}+\sqrt{\frac{\pi}{2}}\frac{\beta S}{(\alpha+\beta)^{3/2}}\right.\\ \left.+{\int}_{0}^{\frac{\beta S}{\alpha+\beta}} \frac{\beta S}{\alpha+\beta}e^{-(\alpha+\beta)u^{2}/2}du\right) \end{array} $$

The analytic expression for first-passage time density, Eq. (67), for arbitrary shape of input (*τ*_*s*_ ≤ *τ*_m_) is achieved:

74 $$\begin{array}{@{}rcl@{}} J(V_{\theta},t)&=&\frac{\gamma}{\alpha+\beta}\left[e^{-\frac{\beta S_{+}^{2}}{2}}+\sqrt{\frac{\pi}{2(\alpha+\beta)}}\beta S_{+}e^{-\frac{\alpha \beta S_{+}^{2}}{2(\alpha+ \beta)}}\left( 1+\text{erf}\left[\frac{\beta S_{+}}{\sqrt{2(\alpha+\beta)}}\right]\right)\right.\\ &&\left.-e^{-\frac{\beta S_{-}^{2}}{2}}-\sqrt{\frac{\pi}{2(\alpha+\beta)}}\beta S_{-}e^{-\frac{\alpha \beta S_{-}^{2}}{2(\alpha+ \beta)}}\left( 1+\text{erf}\left[\frac{\beta S_{-}}{\sqrt{2(\alpha+\beta)}}\right]\right)\right] \end{array} $$

where *γ* = *γ*(*t*,*t*^∗^),*α* = *α*(*t* − *t*^∗^), *β* = *β*(*t*^∗^) and *S*_±_ = *S*_±_(*t*,*t*^∗^) are given by Eq. (68).

To obtain a clearer picture, we combine above definitions and suggest new variables as:

75 $$\begin{array}{@{}rcl@{}} \kappa(t,t^{*}) & \,=\, & {\frac{1\,-\,r(t)^{2}}{1\,-\,r(t\,-\,t^{*})^{2}}},\\ \omega(t,t^{*}) & \,=\, & \frac{r(t\,-\,t^{*})^{2}(1\,-\,r(t^{*})^{2})}{(1\,-\,r(t)^{2})^{3}},\\ \varphi_{\pm}(t,t^{*}) & \,=\, & {\sqrt{\frac{\tau_{\mathrm{m}}V_{\mathrm \theta}^{2}}{D (1\,-\,r(t^{*})^{2})}}} \left\{\pm r(t^{*})\,-\,{{\int}_{0}^{t}} \frac{ds}{\tau_{\mathrm{m}}}\frac{\Delta I(s, t^{*})}{V_{\theta}}\right\}, \end{array} $$

and the first-passage time reads:

76 $$\begin{array}{@{}rcl@{}} J(V_{\theta},t)\,=\,\frac{\sqrt{\kappa \omega}}{\pi \tau_{\mathrm{m}}} \left\{ \exp\left( \!-\frac{\varphi_{+}^{2}}{2}\right)\left[1\,+\,\sqrt{\frac{\pi}{2 \kappa}}\varphi_{+}\exp\left( \frac{\varphi_{+}^{2}}{2 \kappa}\right)\right.\right.\\\left.\!\times\!\left( 1+\text{erf}\left( \frac{\varphi_{+}}{\sqrt{2 \kappa}}\right)\right)\right]\\ \!-\exp\left( -\frac{\varphi_{-}^{2}}{2}\right)\left[1\,+\,\sqrt{\frac{\pi}{2 \kappa}}\varphi_{-}\exp\left( \frac{\varphi_{-}^{2}}{2 \kappa}\right)\right.\\\left.\left.\!\times\!\left( 1\,+\,\text{erf}\left( \frac{\varphi_{-}}{\sqrt{2 \kappa}}\right)\right)\right] \right\}, \end{array} $$

where $\text {erf}(x)=(2/\sqrt {\pi }){{\int }_{0}^{x}} \exp (-t^{2})dt$ .

## Appendix V: Probability of spiking after input arrival starting from the stationary distribution

Prior to input arrival at *t* = 0, membrane potential of the neuron has the stationary distribution (Brunel and Hakim 1999; Brunel 2000):

77 $$\begin{array}{@{}rcl@{}} P_{s}{\kern-.5pt}({\kern-.5pt}V{\kern-.5pt})\! &=&\! \frac{\lambda \tau_{\mathrm{m}}^{2}}{D}{\int}_{V}^{\bar{I}}{\Theta}(V_{s}-V_{r}) \\ &&\! \times \exp\! \left[\frac{-\tau_{\mathrm{m}}}{2 D}\left( {\kern-.5pt}V^{2}{\kern-.5pt}-{\kern-.5pt}{V_{s}^{2}}{\kern-.5pt}-{\kern-.5pt}2\bar{I}(V{\kern-.5pt}-{\kern-.5pt}V_{s}){\kern-.3pt}\right)\right]{\kern-.3pt}dV_{s}, \end{array} $$

Where Θ(*V* ) is the Heaviside step function (Θ(*V* ) = 1when *V* > 0 and otherwise Θ(*V* ) = 0), *λ* is the mean firing rate ($\lambda =({\int }_{0}^{\infty } s J_{0}(V_{\theta },s) \, ds)^{-1} $), *V*_*r*_ is the resting voltage which is zero here and $\bar {I}$ is the mean current which is equal to *V*_*𝜃*_. The stationary distribution is also represented by Eq. (36) at the threshold regime which is the case of our study here. When the square signaling input (see Eq. (3)) arrives at time origin, the probability density of trajectories evolves as:

78 $$\begin{array}{@{}rcl@{}} P(V,\tau)\,=\,\!\left\{\begin{array}{l} \!{\int}_{V_{0}=-\infty}^{V_{\theta}} G(V,\tau;V_{0},0) P_{\mathrm{s}}\left( V_{0}\,-\, {\frac{A}{\tau_{\mathrm{m}}}\!\times\!\frac{\tau}{\Delta t}}\right) d V_{0}. {\quad}\!\!0 \!\leq\! \tau \!\leq\! {\Delta} t,\\ \\ \!{\int}_{V_{0}=-\infty}^{V_{\theta}} G(V,\tau;V_{0},0) P_{\mathrm{s}}\left( V_{0}\,-\, {\frac{A}{\tau_{\mathrm{m}}}}\right) d V_{0}. {\quad\quad\quad} \!\!{\Delta} t \!<\! \tau. \end{array}\right. \end{array} $$

The first passage time, Eq. (25), at time *τ* starting from the stationary distribution represents the trajectories that reach the threshold at that time and it reads:

79 $$\begin{array}{@{}rcl@{}} f(\tau)\,=\,\! \left\{\begin{array}{l}\!-\frac{D}{\tau_{\mathrm{m}}^{2}} {\int}_{V_{0}=-\infty}^{V_{\theta}}\tilde{G}(V,\tau,V_{0},0) P_{\mathrm{s}}\left( V_{0}\,-\, {\frac{A}{\tau_{\mathrm{m}}}\!\times\!\frac{\tau}{\Delta t}}\right) d V_{0}. {\quad}\!\!0 \!\leq\! \tau \!\leq\! {\Delta} t,\\ \\ \!-\frac{D}{\tau_{\mathrm{m}}^{2}}{\int}_{V_{0}=-\infty}^{V_{\theta}} \tilde{G}(V,\tau;V_{0},0) P_{\mathrm{s}}\left( V_{0}\,-\, {\frac{A}{\tau_{\mathrm{m}}}}\right) d V_{0}. {\qquad\quad} \!\!{\Delta} t \!<\! \tau, \end{array}\right. \end{array} $$

where $\tilde {G}(V,t,V_{0},t_{0})$ is given by Eq. (29). In the limit of Δ*t* → 0, the square input looks like delta function (*δ*(*τ*)). At the arrival time of delta function with amplitude $\frac {A}{\tau _{\mathrm {m}}}$, all the trajectories that have the potential $V \geq V_{\theta }-\frac {A}{\tau _{\mathrm {m}}}$ in the stationary state reach the threshold and spike. So the Eq. (79) simply is:

80 $$\begin{array}{@{}rcl@{}} f(\tau)&\,=\,& \delta(\tau){\int}_{V_{0} =V_{\theta}- \frac{A}{\tau_{\mathrm{m}}}}^{V_{\theta}}P_{\mathrm{s}}(V_{0}) d V_{0}\\ &&\!-\frac{D}{\tau_{\mathrm{m}}^{2}}{\int}_{V_{0}=-\infty}^{V_{\theta}} \tilde{G}(V,\tau;V_{0},0) P_{\mathrm{s}}\left( V_{0}\,-\, \frac{A}{\tau_{\mathrm{m}}}\right) d V_{0}. \end{array} $$

The form of Eq. (79) lets us use our suggestion used before in Eq. (30) once more and extend the result in Eq. (79) for an arbitrary shape of signaling input as:

81 $$\begin{array}{@{}rcl@{}} f(\tau)&=& -\frac{D}{\tau_{\mathrm{m}}^{2}}{\int}_{V_{0}=-\infty}^{V_{\theta}} \tilde{G}(V,\tau;V_{0},0)\\ &&\times P_{\mathrm{s}}\left( V_{0}- {{\int}_{0}^{t}}\frac{\Delta I(s,t^{*})}{\tau_{\mathrm{m}}} ds\right) d V_{0}. \end{array} $$

Figure 6b(inset) shows Eq. (81) matches well with Eq. (34).

## Appendix VI: Fisher information when the last postsynaptic spike-time is known

Here, we suppose that the decoder has access to the last spike of postsynaptic neuron, and find the Fisher information by inserting Eq. (28) instead of *f*(*τ*)in Eq. (38). This function helps us to investigate more about the sensitivity of the first passage time to the amplitude of signaling input when the precise time of signaling input is known with respect to last postsynaptic spike. Figure 9 shows the Fisher information as a function of signaling input’s amplitude (*A*) and presynaptic spike time (*t*^∗^). When *t*^∗^occurs immediately after the last spike of postsynaptic neuron, a strong amplitude of the input needs to be added for the voltage to reach the threshold and generates a postsynaptic neuron’s spike. Since the noise has a small effect on voltage trajectory, the Fisher information is maximized. Nevertheless, when *t*^∗^ occurs with delay, noise and mean driving current are added to voltage trajectory and a smaller amount of signaling input is sufficient to reach the threshold; it leads to a local maximum of Fisher information. To find out about the efficient amount of amplitude in each *t*^∗^ that maximizes the Fisher information, we present a simple picture. Roughly, the maximum of Fisher information occurs when *∂**J*(*V*_*𝜃*_,*t*)/*∂**A* is maximum; this corresponds to an *A* which is strong enough to make the postsynaptic neuron to fire, immediately upon presynaptic neuron’s spike. However, If we increase *A* more than this, *J*(*V*_*𝜃*_,*t*) does not significantly increase; this means that *∂**J*(*V*_*𝜃*_,*t*)/*∂**A* would decrease or even vanish, for a larger *A*. We locate such an *A*, it corresponds to an amplitude which makes most of the trajectories to reach the threshold. The trajectories, corresponding to various possible states of the neuron, are all around a mean trajectory, $V(t)=V_{\theta } (1-e^{-t/\tau _{\mathrm {m}}})$. The background noise widens this area, $\sqrt {\frac {D}{\tau _{\mathrm {m}}}(1-e^{-t/\tau _{\mathrm {m}}})}$; thus the amplitude which can make most of the trajectories to spike, should satisfy:

82 $$\begin{array}{@{}rcl@{}} V_{\theta} (1-e^{-t^{*}/\tau_{\mathrm{m}}})-\sqrt{\frac{D}{\tau_{\mathrm{m}}}(1-e^{-t^{*}/\tau_{\mathrm{m}}})}+\frac{A}{\tau_{\mathrm{m}}}=V_{\theta}, \end{array} $$

which shows a relation between *A* and *t*^∗^:

83 $$\begin{array}{@{}rcl@{}} \frac{A}{\tau_{\mathrm{m}}V_{\theta}}=e^{-t^{*}/\tau_{\mathrm{m}}}+\sqrt{\frac{D}{\tau_{\mathrm{m}} V_{\mathrm{\theta}}^{2}}(1-e^{-t^{*}/\tau_{\mathrm{m}}})}. \end{array} $$

The dashed line in Fig. 9, show Eq. (83) that has good agreement with circles (amplitude maximizing Fisher information as a function of *t*^∗^) as far as *t*^∗^≤ *t*_max_. The threshold nonlinearity and image method disturb this simple picture for *t*^∗^ > *t*_max_. The deviation between the dashed line and circles shows how threshold makes the picture complicated. The solid line arises from calculating a voltage *V* which satisfies *∂*^2^*P*_0_(*V*,*t*)/*∂**V*^2^ = 0(see Eq. (7)); it shows a very good agreement with the place of maximized Fisher information.

Figure 9 gives us a hint why we observe two maximums in the Fisher information for the low level of the noise in Fig. 6. The left panel of Fig. 9 shows the Fisher information for low scaled diffusion (*D*/(*τ*_m_*V**𝜃*2) = 0.004). In this figure, the Fisher information is maximized at stronger amplitude for earlier *t*^∗^(*t*^∗^≤ *t*_*m**a**x*_), but at weaker amplitude for later *t*^∗^(*t*^∗^≥ *t*_*m**a**x*_). It is likely that we observed these two peaks in Fig. 6a for small diffusions because this result (see Eq. (38)) is obtained roughly by averaging over *t*^∗^ for each amount of signaling input’s amplitude in Fig. 9.
